# Supplementary material for: Antiseizure medication use during pregnancy and children’s neurodevelopmental outcomes
Source: Nat Commun. 2024 Nov 15;15:9640. doi: 10.1038/s41467-024-53813-1 (PMC11568279; doi:10.1038/s41467-024-53813-1)
Supplement: Supplementary file 1 — Supplementary Information [file 41467_2024_53813_MOESM1_ESM.pdf]

Supplementary Material

Antiseizure Medication Use During Pregnancy and Children's Neurodevelopmental Outcomes

Contents

Supplementary Background 3

Synthesis of recent large-scale studies of children's neurodevelopmental outcomes........ 3

Carbamazepine consistency across studies 3

Supplementary Methods 6

STROBE Statement—checklist 6

Data sources 10

CPRD GOLD (UK) 10

DOHaD (Sweden) 11

Uncertain pregnancies in the CPRD pregnancy register 13

Exposure: maternal antiseizure medication 14

Prescription data cleaning procedure – CPRD (UK) 14

Definition of "other ASMs" 15

Outcome: offspring neurodevelopmental conditions 16

Eligibility for linkage to Hospital Episode Statistics data – CPRD (UK) 16

Covariates 17

Antiseizure medication (ASM) indication definitions 17

Other covariate definitions 19

Missing data 22

CPRD GOLD (UK) 22

DOHaD (Sweden) 22

Statistical analysis 22

Follow-up time 22

Supplementary Results 23

Supplementary Tables 23

Table S1 – Country and ASM specific exposure counts and follow up time 23

Table S2 – Primary Analysis: Country specific adjusted marginal risk results 24

Table S3 – Primary Analysis: Pooled adjusted marginal risk results 25

Table S4 – Primary Analysis: Country specific risk difference results 26

Table S5 – Primary Analysis: Pooled risk difference (relative to no ASM) results 27

Table S6 – Primary Analysis: Country specific and combined hazard ratio results 28

Table S7 – Heterogeneity Tests: Wald tests of hazard ratio comparisons 29

Table S8 – Discordant Sibling Analysis: Country specific and combined results 31

Table S9 – Active Comparator Analysis: Country specific and combined results 32

Table S10 – Indication Stratified Analysis: Counts of exposed and exposed with each outcome in each country according to indication 33

Table S11 – Indication Stratified Analysis: Country specific adjusted marginal risk results........ 35

Table S12 – Indication Stratified Analysis: Pooled adjusted marginal risk at age 12 results....... 36

Table S13 – Sensitivity Analysis: Pooled hazard ratio results 37

Table S14 – Sensitivity Analysis: Comparison of primary analysis models including and excluding vomiting or antiemetics as a covariate 38

Table S15 – Counts of antiseizure medications included in the polytherapy category 39

Supplementary Figures........................ 40

Figure S1 – Flowchart of cohort derivation for CPRD (UK) 40

Figure S2 – Flowchart of cohort derivation for DOHaD (Sweden) 41

Figure S3 – Primary Analysis: Pooled risk difference results relative to no ASM at age 12......... 42

Figure S4 – Primary Analysis: Country specific risk difference results for autism relative to no ASM 43

Figure S5 – Primary Analysis: Country specific risk difference results for intellectual disability relative to no ASM........................ 44

Figure S6 – Primary Analysis: Country specific risk difference results for ADHD relative to no ASM 45

Figure S7 – Primary Analysis: Country specific hazard ratio results........................ 46

Figure S8 – Discordant Sibling Analysis: Country specific within-family hazard ratio results...... 47

Figure S9 – Active Comparator Analysis: Country specific hazard ratio results 48

Figure S10 – Indication Stratified Analysis: Pooled adjusted marginal risk at age 12 results 49

Figure S11 – Indication Stratified Analysis: Pooled hazard ratio results 50

Figure S12 – Indication Stratified Analysis: Country specific hazard ratio results for epilepsy... 51

Figure S13 – Indication Stratified Analysis: Country specific hazard ratio results for psychiatric indications 52

Figure S14 – Indication Stratified Analysis: Country specific hazard ratio results for somatic indications 53

References........................ 54

Supplementary Background

Synthesis of recent large-scale studies of children's neurodevelopmental outcomes

There have been a series of large-scale recent efforts dedicated to studying specific ASMs in pregnancy and children's neurodevelopmental/cognitive outcomes (see Supplementary Background Table 1), several of which have relied on routinely collected data. Each of the studies has specific strengths and weaknesses, with small or larger differences in approaches. These differences include variations in exposure (e.g., dispensations or self-report) and outcome definitions (e.g., clinical diagnosis or standardized tests), populations (e.g., total population or mothers with epilepsy), and analytical approaches (e.g., time-to-event or contrasts in expected distributions). For studies on clinically diagnosed neurodevelopmental outcomes, the number of children with neurodevelopmental conditions exposed to specific ASMs typically remains limited to tens, despite utilizing extensive electronic health records, insurance claims, or nationwide registries.

Carbamazepine consistency across studies

Since we found an increased risk associated with carbamazepine, which has not been widely reported before, we conducted a rapid synthesis of estimates from previous studies to assess if our estimate deviated from literature expectations. The populations in these studies, including ours, overlap, and therefore, one should not interpret a pooled meta-analyzed estimate across studies. However, to illustrate the quantitative consistency across studies, we performed a fixed-effects meta-analysis. We find that the estimates are consistent across studies for autism and ADHD (I^2 = 0%) (see Supplementary Background Figure 1). For intellectual disability, there is some variation (I^2 = 75.4%), which appears to be explained by the large effect found in Denmark by Daugaard et al.[1]. However, our findings are consistent with those of Bjørk et al.[2] and Dreier et al.[3], although ours is statistically significant due to the larger number of exposed cases. Finally, we note that Ren et al.[4] found that children exposed to carbamazepine in pregnancy performed worse on standardized school tests at grade 9.

Supplementary Background Table 1 – Recently published studies of antiseizure medications in pregnancy and offspring neurodevelopmental conditions or related outcomes

Author(s) (year)	Country	Study design	Exposure(s)	Outcome(s)	Sample size	Finding(s)	Strength(s)	Limitation(s)
Hernández-Díaz et al. (2024)	United States	Insurance claims	Topiramate, valproate or lamotrigine in second half of pregnancy	Autism	4 292 539	Valproate associated with autism, but not topiramate and lamotrigine	Large sample size, detailed sensitivity analysis	Only studies autism and has large loss to follow-up (resulting in a median of 2 years of follow-up)
Cohen et al. (2023)	United States	Clinical cohort	Third trimester antiseizure medication blood concentration	Adaptive behavior outcomes, and score-defined autism, ADHD, and intellectual disability	386	No difference between exposed and control patients, but higher blood concentration was associated with poorer adaptive behavior	Objective measure of exposure, detailed data on each participant	Small sample size, unable to study clinical outcomes, only stratified on lamotrigine and levetiracetam
Knight et al. (2023)	United Kingdom	Phone interviews with topiramate users	Topiramate	Adaptive behavior outcomes and autism	21[a]	Topiramate was associated with poorer adaptive behavior and autism	Detailed patient contact	Retrospective and no "control-group" (compared to expected distributions)
Daugaard et al. (2020)	Denmark	Registry-based	Several antiseizure medications	Intellectual Disability and "Delayed Childhood Milestones"	913 302	Valproate, carbamazepine, and oxcarbazepine were associated with increased risk of intellectual disability and delayed childhood milestones	Examines a broad range of antiseizure medications, general population	Few exposed cases
Bjørk, et al. (2022)	Denmark, Finland, Iceland, Norway, & Sweden	Registry-based	Several antiseizure medications	Autism and intellectual disability	4 702 774	Topiramate, valproate, and several duo therapies were associated with increased risks of neurodevelopmental disorders	Examines a broad range of antiseizure medications, general population	Few exposed cases, and no reported adjustment for epilepsy as an indication
Dreier et al. (2023)	Denmark, Finland, Iceland, Norway, & Sweden	Registry-based of women with epilepsy	Several antiseizure medications	Childhood- and Adolescence-Onset Psychiatric Disorders	38 661	Topiramate and levetiracetam were associated with ADHD	Examines a broad range of antiseizure medications	Restricted to women with epilepsy
Ren et al. (2023)	Denmark	Registry-based	Carbamazepine	Academic performance (ninth-grade exit examination)	370 859	Carbamazepine was associated with poorer academic performance	Does not rely on 'diagnostic' detection	Unclear clinical relevance of the outcome
Wiggs et al. (2020)	Sweden	Registry-based of women with epilepsy	"Any antiseizure medication" and valproate, lamotrigine, and carbamazepine	Autism and ADHD	14 614	Valproate was associated with autism and ADHD. Carbamazepine was not statistically significant (albeit elevated risk)	Use of older exposure data to increase the effective sample size	Restricted to women with epilepsy

[a] Topiramate exposed children.

Supplementary Background Figure 1 – Fixed-effects meta-analysis of carbamazepine estimates from recently published studies. The effect measure is the fully adjusted relative risk (mostly hazard ratio) in the respective study, with 95% confidence intervals.

Outcome and Study	Exposed cases	Population	Data	Effect measure (95% CI)	% Weight
**Autism**					
Bjørk, et al. (2022)	41	General pop	DK, FIN, ICE, NOR, SWE	1.36 (1.00, 1.85)	10.37
Bjørk, et al. (2022)	26	Epilepsy	DK, FIN, ICE, NOR, SWE	0.94 (0.60, 1.46)	4.96
Dreier et al. (2023)	42	Epilepsy	DK, FIN, ICE, NOR, SWE	1.03 (0.70, 1.51)	6.64
Wiggs et al. (2020)	46	Epilepsy	SWE	1.26 (0.88, 1.79)	7.78
Current study	157	General pop	SWE & UK	1.25 (1.05, 1.48)	33.30
Current study	115	Epilepsy	SWE & UK	1.21 (0.98, 1.50)	21.65
Current study	40	Psychiatric	SWE & UK	1.37 (0.99, 1.90)	9.23
Current study	29	Somatic	SWE & UK	1.46 (0.98, 2.19)	6.07
Subgroup, IV (I^2 = 0.0%, p = 0.803)				1.24 (1.12, 1.37)	100.00
**Intellectual Disability**					
Daugaard et al. (2020)	15	General pop	DK	3.84 (2.32, 6.38)	7.34
Bjørk, et al. (2022)	20	General pop	DK, FIN, ICE, NOR, SWE	2.01 (1.45, 2.79)	17.52
Bjørk, et al. (2022)	36	Epilepsy	DK, FIN, ICE, NOR, SWE	0.83 (0.49, 1.42)	6.63
Dreier et al. (2023)	36	Epilepsy	DK, FIN, ICE, NOR, SWE	1.11 (0.73, 1.70)	10.50
Current study	67	General pop	SWE & UK	1.30 (1.01, 1.69)	28.33
Current study	≈56	Epilepsy	SWE & UK	1.46 (1.08, 1.97)	20.78
Current study	7	Psychiatric	SWE	0.75 (0.37, 1.54)	3.69
Current study	≈13	Somatic	SWE & UK	1.88 (1.03, 3.42)	5.21
Subgroup, IV (I^2 = 75.4%, p = 0.000)				1.48 (1.29, 1.70)	100.00
**ADHD**					
Wiggs et al. (2020)	81	Epilepsy	SWE	1.18 (0.91, 1.52)	9.93
Dreier et al. (2023)	89	Epilepsy	DK, FIN, ICE, NOR, SWE	1.05 (0.81, 1.37)	9.47
Current study	270	General pop	SWE & UK	1.07 (0.94, 1.21)	41.01
Current study	201	Epilepsy	SWE & UK	1.04 (0.89, 1.22)	26.28
Current study	50	Psychiatric	SWE & UK	1.05 (0.79, 1.40)	7.98
Current study	≈33	Somatic	SWE & UK	0.96 (0.68, 1.37)	5.33
Subgroup, IV (I^2 = 0.0%, p = 0.957)				1.06 (0.98, 1.15)	100.00
Heterogeneity between groups: p = 0.000					

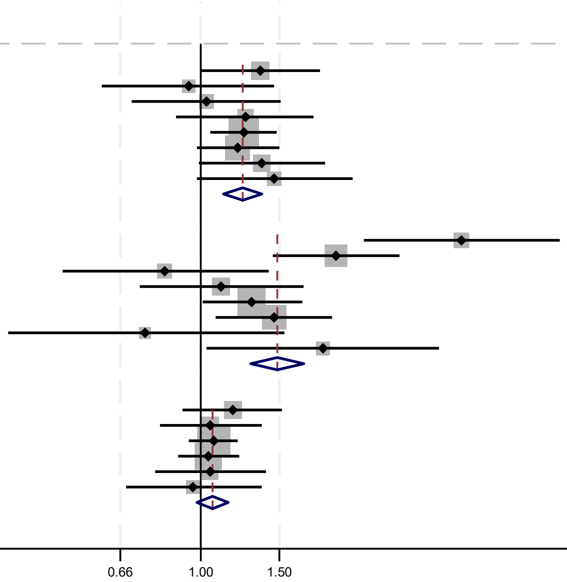

Outcome and Study	Exposed N	Population	Data	Z-score (95% CI)	% Weight
Ren et al. (2023)	226	Epilepsy	DK	-0.11 (-0.23, 0.01)	43.36
Ren et al. (2023)	290	General pop	DK	-0.17 (-0.28, -0.07)	56.64
**Mathematics 9th grade test**					
Subgroup, IV (I^2 = 0.0%, p = 0.461)				-0.14 (-0.22, -0.06)	100.00
Ren et al. (2023)	226	Epilepsy	DK	-0.08 (-0.18, 0.03)	45.01
Ren et al. (2023)	290	General pop	DK	-0.14 (-0.24, -0.05)	54.99
**Danish 9th grade test**					
Subgroup, IV (I^2 = 0.0%, p = 0.406)				-0.11 (-0.18, -0.04)	100.00
Heterogeneity between groups: p = 0.566					

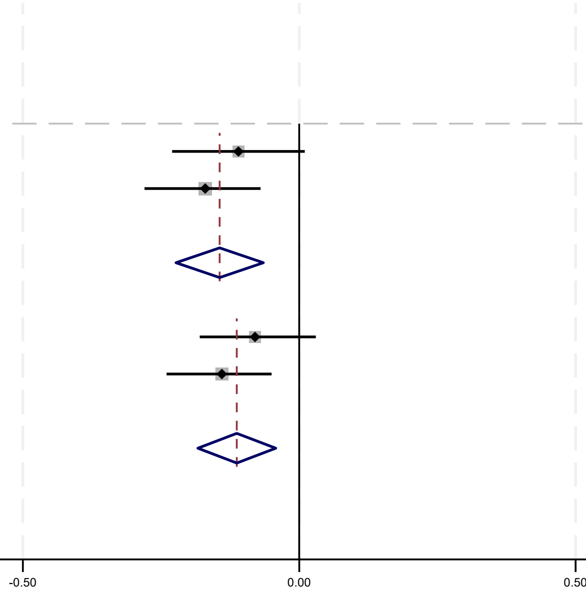

Supplementary Methods

STROBE Statement—checklist

	Item No.	Recommendation	Relevant section from manuscript
**Title and abstract**	1	(*a*) Indicate the study's design with a commonly used term in the title or the abstract	Title
		(*b*) Provide in the abstract an informative and balanced summary of what was done and what was found	Abstract
**Introduction**			
Background/rationale	2	Explain the scientific background and rationale for the investigation being reported	Introduction (paragraph 1-4)
Objectives	3	State specific objectives, including any prespecified hypotheses	Introduction (paragraph 5)
**Methods**			
Study design	4	Present key elements of study design early in the paper	Methods (paragraph 1)
Setting	5	Describe the setting, locations, and relevant dates, including periods of recruitment, exposure, follow-up, and data collection	Methods (Data sources)
Participants	6	(*a*) *Cohort study*—Give the eligibility criteria, and the sources and methods of selection of participants. Describe methods of follow-up	Methods (Study population)
		(*b*) *Cohort study*—For matched studies, give matching criteria and number of exposed and unexposed	NA
Variables	7	Clearly define all outcomes, exposures, predictors, potential confounders, and effect modifiers. Give diagnostic criteria, if applicable	Methods (Exposure: Maternal antiseizure medication section; Outcome: Children's neurodevelopmental conditions; Covariates); Supplementary Methods

	Item No.	Recommendation	Relevant section from manuscript
Data sources/ measurement	8*	For each variable of interest, give sources of data and details of methods of assessment (measurement). Describe comparability of assessment methods if there is more than one group	Methods (Data sources section; Exposure: Maternal antiseizure medication section; Outcome: Children's neurodevelopmental conditions; Covariates section); Supplementary Methods
Bias	9	Describe any efforts to address potential sources of bias	Methods (Primary analysis; Secondary analysis; Sensitivity analysis)
Study size	10	Explain how the study size was arrived at	Methods (Study Population); Supplementary Figure S1 & S2
Quantitative variables	11	Explain how quantitative variables were handled in the analyses. If applicable, describe which groupings were chosen and why	Methods (Covariates); Supplementary Methods (Covariates)
Statistical methods	12	(*a*) Describe all statistical methods, including those used to control for confounding	Methods (Primary analysis; Secondary analysis)
		(*b*) Describe any methods used to examine subgroups and interactions	Methods (Primary analysis)
		(*c*) Explain how missing data were addressed	Supplementary Methods (Missing data section)
		(*d*) *Cohort study*—If applicable, explain how loss to follow-up was addressed	Methods (Primary analysis); supplementary methods
		(*e*) Describe any sensitivity analyses	Methods (Secondary analysis; Sensitivity analysis)
**Results**			

	Item No.	Recommendation	Relevant section from manuscript
Participants	13*	(a) Report numbers of individuals at each stage of study—eg numbers potentially eligible, examined for eligibility, confirmed eligible, included in the study, completing follow-up, and analysed	Methods (Study Population); Supplementary Figure S1 & S2
		(b) Give reasons for non-participation at each stage	Supplementary Figure S1 & S2
		(c) Consider use of a flow diagram	Supplementary Figure S1 & S2
Descriptive data	14*	(a) Give characteristics of study participants (eg demographic, clinical, social) and information on exposures and potential confounders	Results-paragraph 1; Table 1
		(b) Indicate number of participants with missing data for each variable of interest	Supplementary Methods (Missing data section); Supplementary Figure S1 & S2
		(c) *Cohort study*—Summarise follow-up time (eg, average and total amount)	Supplementary Table S1
Outcome data	15*	*Cohort study*—Report numbers of outcome events or summary measures over time	Results (Paragraph 1)
Main results	16	(*a*) Give unadjusted estimates and, if applicable, confounder-adjusted estimates and their precision (eg, 95% confidence interval). Make clear which confounders were adjusted for and why they were included	Results (Primary analyses: ASM use and …); Supplementary Tables S2-5 and Figures S3-7
		(*b*) Report category boundaries when continuous variables were categorized	Table 1
		(*c*) If relevant, consider translating estimates of relative risk into absolute risk for a meaningful time period	Results (ASM use and …); Figure 1
Other analyses	17	Report other analyses done—eg analyses of subgroups and interactions, and sensitivity analyses	Results (Sibling analyses …); Figure 3
**Discussion**			
Key results	18	Summarise key results with reference to study objectives	Discussion (Paragraph 1)

	Item No.	Recommendation	Relevant section from manuscript
Limitations	19	Discuss limitations of the study, taking into account sources of potential bias or imprecision. Discuss both direction and magnitude of any potential bias	Discussion (Strength and limitations)
Interpretation	20	Give a cautious overall interpretation of results considering objectives, limitations, multiplicity of analyses, results from similar studies, and other relevant evidence	Discussion
Generalisability	21	Discuss the generalisability (external validity) of the study results	Discussion (Strength and limitations)
**Other information**			
Funding	22	Give the source of funding and the role of the funders for the present study and, if applicable, for the original study on which the present article is based	Disclosures (Funding statement)

Data sources

CPRD GOLD (UK)

Over 98% of the UK population are registered with a general practitioner (GP) and the GP is the gatekeeper of healthcare in the UK.[5] CPRD GOLD collates routinely collected anonymised health records from GP practices agreeing to provide data; these practices have data from around 6.9% of the UK population (as of 2015, though coverage has since increased) and the patients are broadly representative of the United Kingdom population in terms of age and sex.[5] Continuous CPRD data are available for each patient since registration at the practice, including diagnoses (recorded using Read codes)[6], prescriptions (recorded using British National Formulary codes), and basic demographic data. We used the CPRD GOLD August 2021 build for our analyses [7]

The CPRD Pregnancy Register lists all pregnancies identified in the CPRD for women aged 11–49 years, with pregnancy episodes identified using an algorithm.[8] Summarised details of the algorithm can be found in Figure 1 of the register profile published by Minassian et al. The register includes pregnancy outcome derived from Read codes and Entity types, and estimates of pregnancy timings (including start and end of pregnancy) from date information accompanying the Read codes and Entity types. Validation work, comparing the Pregnancy Register against linked electronic maternity records in HES, has indicated overall good agreement, suggesting most pregnancies are well captured in the register.[8] Pregnancies in the register were identified with 91% sensitivity as compared to a gold standard of hospital deliveries recorded in HES and 95% of delivery dates in the register were within 2 days or dates in HES. We used the July 2020 build of the CPRD GOLD Pregnancy Register.

The CPRD Mother-Baby link allows for linkage between individuals within the same family as a result of a practice specific family identifier[9]. This linkage allowed us to identify prescriptions/covariates in the mothers and diagnoses of neurodevelopmental outcomes in the live-born child. We used the August 2021 build of the Mother-Baby link for this study.

We supplemented the primary care data of CPRD GOLD with the linked Hospital Episode Statistics (HES) database. HES data covers admitted patient/inpatient care (from 1997), outpatient and emergency care data (from 2003) for approximately 80% of the English practices included in CPRD. HES data contains diagnoses (coded using international classification of diseases, 10th revision) but not hospital-based prescriptions.[5] The data build used for linked HES data was set 21[10-12].

We used linked Index of Multiple Deprivation (IMD) data, which is patient[13] or practice[14] level deprivation data. IMD score is calculated by mapping patients' or practice postcode to geographical areas with predefined deprivation scores; data from several indicators, chosen to cover a range of economic, social, and housing issues, are combined into a single deprivation score.[5] Patient level IMD

was available for most practices in England (corresponding to 77% of patients in England) and practice level IMD was available for all practices in Northern Ireland, Scotland and Wales. Patient level IMD was used where available and practice level IMD was used otherwise – 53% of patients had patient level IMD data available. The IMD score is only provided at a single time point by CPRD for each country: 2015 for England, 2017 for Northern Ireland, 2016 for Scotland and 2014 for Wales. Due to changes in the sociodemographic make up of areas over time, the IMD score may not accurately reflect deprivation in the mother's area of residence at the time of pregnancy, with less accuracy the further the pregnancy was from the time of IMD recording.

Information on patient date of death was obtained from the Office for National Statistics (ONS) death certificate data[15].

DOHaD (Sweden)

The Swedish Developmental Origins of Health and Disease (DOHaD) study is a large-scale project that integrates multiple Swedish registries to advance research on pregnancy-related issues, with a particular emphasis on drug safety during pregnancy[16]. Utilizing the Swedish personal identification numbers[17]—assigned to every resident at birth or upon emigration—the study directly links data across Swedish registries with the help of Swedish registry authorities/holders. While the DOHaD study itself does not necessitate independent validation, its findings are inherently dependent on the accuracy and reliability of the underlying registries, which are detailed further below.

Swedish antenatal and obstetric care is publicly funded; almost all pregnant women attend antenatal care regularly and give birth in a hospital. The Swedish Medical Birth Register covers virtually all children born in Sweden (97% to >99% for every year between 1973 and 2020)[18]. While it is technically possible to report planned or unplanned home deliveries, these likely account for the small amount of missing data in the registry[18]. We included all identifiable pregnancies between July 1, 1995, and December 31, 2020.

Along with antenatal, obstetric, and neonatal data, the Swedish Medical Birth Register also contains antenatal records of medication use[18]. Early drug exposure information was prospectively collected during the first antenatal visit (typically occurring at 8-10 weeks of gestation), where midwives conducted structured interviews and examinations, recording prescription and over-the-counter medications[18]. Additional medication use throughout pregnancy is documented by midwives and doctors within antenatal records. These data are later translated into Anatomical Therapeutic Chemical (ATC) codes by the National Board of Health and Welfare. We obtained this data for all pregnancies between July 1, 1995, and December 31, 2020.

From 2005 onwards, we supplemented antenatal reporting of medication use with prescription data from the nationwide Swedish Prescribed Drug registry[19]. To account for prescriptions lasting into pregnancy, we also include any prescriptions made up to 30 days before pregnancy. This registry contains all drug dispensations in Sweden since 2005, recording ATC codes, dates of dispensations, and other related information.

We follow all children through the National Patient Register[20] using unique IDs assigned to each child born in Sweden[17]. The registry covers all inpatient care delivered in Sweden and, from approximately 2005, specialized outpatient care[20]. The registry is generally considered to have high validity and is routinely used for research purposes[20]. All diagnoses are recorded using ICD codes, and dates are marked. We collect any available data from the registry between July 1, 1995, and December 31, 2021, for all identified children. The registry was also employed to identify maternal covariates and drug indications.

In addition to the aforementioned data sources, we acquire income and education data from the Longitudinal Integrated Database for Health Insurance and Labour Market Studies (LISA)[21], which encompasses socioeconomic and demographic data on all Swedish residents since 1990. This data is mandated by law, primarily for administrative purposes[21].

Uncertain pregnancies in the CPRD pregnancy register

A substantial proportion of pregnancies in the CPRD GOLD Pregnancy Register are uncertain, either having no identified outcome, or they overlap ("conflict") with other pregnancies. Ignoring these records potentially excludes periods when women were pregnant. Work by the CPRD Pregnancy Register developers[22] has investigated the frequency of various scenarios explaining unknown/conflicting pregnancies, and in line with their advice for drug safety studies we performed the following actions:

1. Utilise linked data to obtain additional outcomes

The CPRD Pregnancy register only utilises data from primary care record. We used HES data (HES APC, HES Maternity, HES Outpatients and HES Procedures) to identify outcomes (delivery, miscarriage, or termination of pregnancy). For deliveries, records within 266 days of pregnancy start plus 4 weeks (38 weeks) were retained, and for early pregnancy losses, records within 140 days from first antenatal record (20 weeks) were retained. HES records were only available for those with linked data (N=596,218 (48.5%)). We used the approach outlined in the CPRD Pregnancy Register validation study.[8]

2. Merging conflicting pregnancies episodes

We merged conflicting episodes which are consistent with the pregnancy being real but split into separate episodes by the rules of the Pregnancy Register algorithm.

3. Excluding episodes which are likely to be derived from historical data

There is evidence to suggest that historical outcomes being recorded by the GP during an ongoing pregnancy may explain a sizeable proportion of the uncertain episodes generated by the algorithm. This can lead to true pregnancies being split by the algorithm and depending on the timing this will either generate an additional episode with outcome missing or two separate episodes with outcomes.

Exposure: maternal antiseizure medication

Prescription data cleaning procedure – CPRD (UK)

Prescription start and end date were used to define the exposure windows. While prescription start date is available within CPRD, prescription end date was estimated by calculating a prescription length equal to daily dose divided by quantity prescribed. The cleaning procedure for daily dose and quantity are prescribed below.

In total there were 1,281,989 ASM prescription records within the study period for 44,725 women. Combining free text and structured data we were able to recover dose units information units (capsules, tablets, milligrams, millilitres, puffs, sachets and suppositories) for all but 5 prescriptions; these were removed.

Daily dose (the number of units prescribed per day) was missing for 296,953 (23%) records and quantity (the total quantity entered by the GP for the prescribed product) was missing for 813 (0.1%) records. For each ASM prescription, implausible values of daily dose and quantity prescribed were identified and set to missing. For daily dose, values of 0 (for any unit including tablets, capsules, milligrams, millilitres or puffs) and 10 or more tablets or capsules per day were considered implausible - 90,593 (7%) records had daily dose equal to 0 and 1447 (0.1%) records for tablets or capsules had a daily dose greater than 10. For quantity prescribed, values of less than 7 (for any unit) were considered implausible - 10,084 (0.8%) records had quantity values set to missing.

A single imputation procedure was then implemented to impute missing values of daily dose, followed by quantity prescribed. For daily dose prescribed, if daily dose was missing, the value was imputed using the modal daily dose within strata of the earliest possible of the following set of variables:

1. Dosage unit, patient, product code and quantity
2. Dosage unit, product code and quantity in any patient
3. Dosage unit and product code in any patient
4. Dosage unit and product name in any patient
5. Dosage unit and drug in any patient

The procedure was then repeated for quantity of medication prescribed with daily dose replaced with quantity and vice versa. The mean (SD) daily dose for non-imputed data was 4.34 (38.30) and median (IQR) =was 2 (2-3). For imputed data the mean (SD) daily dose was 3.14 (10.93) and median (IQR) was 2 (2-3). The mean (SD) quantity for non imputed data was 86.90 (89.65) and median (IQR) was 56 (60-112). For imputed data the mean (SD) quantity was 53.62 (56.17) and median (IQR) was 28 (18-84).

Definition of “other ASMs”

The following ASMs were included in the “other ASM” category: brivaracetam, eslicarbazepine, ethosuximide, felbamate, lacosamide, oxcarbazepine, perampanel, phenobarbital, primidone, retigabine, rufinamide, stiripentol, sulthiame, tiagabine, vigabatrin, zonisamide, clonazepam, beclamide, mesuximide, phenacemide, ethotoin, pheneturide, carisbamate, cenobamate, barbexaclone, ethadione, progabide, clobazam.

Outcome: offspring neurodevelopmental conditions

Eligibility for linkage to Hospital Episode Statistics data – CPRD (UK)

Of the total cohort of 518,047 children in the UK, 273,018 (52.7%) were eligible for linkage to HES data. Where children were eligible for data linkage, diagnoses made using either Read or ICD-10 codes were used to indicate presence of the outcome. Where children were ineligible for data linkage, only Read code diagnoses were used to indicate presence of the outcome. Details of the numbers and proportions diagnosed using Read codes (total sample) and ICD-10 codes (sample eligible for HES linkage only) are presented below according to exposure to any ASM during the pregnancy period. The proportion diagnosed with each neurodevelopmental condition was similar in the total cohort and the cohort eligible for linkage to HES data, though a greater proportion were diagnosed using Read codes than were diagnosed using ICD-10 codes.

	**Total cohort – outcome identified using Read or ICD-10 codes (N%)**		**Cohort eligible for HES linkage - outcome identified using Read or ICD-10 codes (N%)**		**Total cohort - outcome identified using Read codes (N%)**		**Cohort eligible for HES linkage - outcome identified using ICD-10 codes (N%)**	
	No ASM use in pregnancy (N=514,066)	ASM use in pregnancy (N=3981)	No ASM use in pregnancy (N=271,253)	ASM use in pregnancy (N=1765)	No ASM use in pregnancy (N=514,066)	ASM use in pregnancy (N=3981)	No ASM use in pregnancy (N=271,253)	ASM use in pregnancy (N=1765)
Autism	7657 (1.49)	104 (2.61)	3846 (1.42)	38 (2.15)	6114 (1.19)	87 (2.19)	2217 (0.82)	25 (1.42)
Intellectual disability	1119 (0.22)	15 (0.38)	731 (0.27)	7 (0.40)	763 (0.15)	11 (0.28)	394 (0.15)	5 (0.28)
ADHD	4794 (0.93)	61 (1.53)	2641 (0.97)	32 (1.81)	3661 (0.71)	42 (1.06)	1140 (0.42)	16 (0.91)

Covariates

Antiseizure medication (ASM) indication definitions

Indications for ASMs were grouped as epilepsy, psychiatric conditions and somatic conditions. Psychiatric conditions included bipolar disorder, generalised anxiety disorder, severe depression, treatment resistant depression and off label psychiatric use of ASM medication. Somatic conditions included neuropathic pain, fibromyalgia, essential tremor, restless leg syndrome and migraine prophylaxis. In Sweden, data protection regulations (i.e., to prevent the identification of individuals due to the rarity of certain diseases) led to the truncation of somatic condition ICD codes by registry holders (e.g., ICD 10: M70-M79.9), resulting in broader definitions than in the UK. The timing for the evidence of indication in relation to pregnancy was recorded and only indications occurring before the start of pregnancy were kept. Read code and ICD-10 code lists can be found in the GitHub repository.

Details of the definition for each indication in each study site are included in the table below:

	CPRD (UK) Definition		DOHaD (Sweden) Definition	
**Indication covariate**	Phenotypes	Identification	Phenotypes	Identification
**Epilepsy**	-	Patients had a record of one of the following:<br>1. A diagnosis of epilepsy in CPRD or HES;<br>a. CPRD: (presence of an epilepsy Read code) OR (two seizure Read codes more than 24 hours apart AND no correlating neurology outpatient appointment within one month of primary care recorded seizure code (as likely duplicates))<br>b. Inpatient HES: presence of an epilepsy ICD code in any diagnostic position OR two seizure ICD codes more than 24 hours apart listed in the first diagnostic position AND the event is in the relevant medical specialty AND emergency admission<br>c. Outpatient HES: Epilepsy ICD code listed in any diagnostic position<br>d. Emergency HES: Two seizure ICD codes more than 24 hours apart<br>2. Prescription of epilepsy-specific ASMs: Epilim, Brivaracetam, Brivaracetam, Eslicarbazepine, Ethosuximide, Felbamate, Fenfluramine, Lacosamide, Levetiracetam, Mesuximide, Oxcarbazepine, Perampanel, Phenobarbital, Phenytoin, Retigabine, Rufinamide, Stiripentol, Sulthiame, Tiagabine, Vigabatrin, Zonisamide, OR;	-	Inpatient or specialized outpatient ICD-10 G40.X diagnosis which has been preceded by a G40.X (ICD-9 345+) or R56.8 (unspecified convulsions).

		Epilepsy-specific co-prescribing on the same day: i) Clobazam AND an ASM OR ii) rectal administration of diazepam AND an ASM OR 3) intranasal administration of Midazolam AND an ASM		
**Psychiatric indications**	Bipolar disorder	Patients had a record of one of the following:<br>1. Read code in CPRD or ICD-10 code in HES (any diagnostic field) for bipolar, anytime prior to pregnancy start date OR;<br>2. Mood-disorder specific co-prescribing (1-Quetiapine and [valproate or lamotrigine or carbamazepine] or 2-lithium AND [valproate or lamotrigine or carbamazepine]) OR;<br>3. The mood disorder-specific ASM Depakote.	Bipolar disorder	Inpatient or specialized outpatient diagnosis: ICD-10: F30, F31; ICD-9: 296A, 296C, 296D 296E.
	Generalised anxiety disorder	An ASM used in psychiatry (valproate or lamotrigine or carbamazepine) along with a Read code in CPRD or ICD-10 code in HES for anxiety (any diagnostic field), anytime prior to pregnancy start date.	Generalised anxiety disorder	ICD-10: F40-F43<br>ICD-9: 300A, 300C, 300D, 308, 309
	Severe depression	An ASM used in psychiatry (valproate or lamotrigine or carbamazepine) along with a READ code in CPRD or ICD-10 code in HES (any diagnostic field) for severe depression, anytime prior to pregnancy start.	Depression	ICD-10: F32-F39<br>ICD-9: 296B, 298A, 300E, 311
	Treatment-resistant schizophrenia	An ASM used in psychiatry (valproate or lamotrigine or carbamazepine) along with READ code in CPRD or ICD-10 code (any diagnostic field) in HES for schizophrenia, anytime prior to pregnancy start.	Other off-label psychiatric use	ICD-10: F00-F99<br>ICD-9 and icd-8: 29.0-29.9, 30.0-30.9, 31.0-31.9
	Other off-label psychiatric use	None of the above psychiatric indications were identified, yet there was co-prescription of antipsychotics, lithium, or antidepressants, alongside an ASM used in psychiatry (valproate or lamotrigine or carbamazepine) in CPRD.		
**Somatic indications**	Neuropathic pain (including diabetic neuropathy) and fibromyalgia	An ASM used in neuropathic pain management (carbamazepine, barbexaclone, gabapentin, pregabalin) alongside either 1) a READ code in CPRD or ICD-10 code in HES (any diagnostic field) for a neuropathic pain disorder, anytime prior to pregnancy start or 2) evidence of codeine co-prescribing.	Migraine	ICD-10: G43.0-G43.9<br>ICD-9 and ICD-8: 346.0-346.9
	Migraine prophylaxis	An ASM used for the prevention of recurrent migraine (topiramate or valproate) along with a READ code in CPRD or ICD-10 code in HES (any diagnostic field) for recurrent migraine, anytime prior to pregnancy start.	Chronic pain	ICD-10: M70.0-M79.9, R52.0-R52.9, G35.0-G35.9, G50.0-G59.9, F45.0-F45.9<br>ICD-9: 350.0-359.9, 723.0-723.9, 724.0-724.9<br>ICD-8: 350.0-358.9
	Restless legs syndrome	The ASM cenobamate is used for the treatment of restless leg syndrome along with a READ code in CPRD or ICD-10 code in HES (any diagnostic field) for restless leg syndrome, anytime prior to pregnancy start.	Diabetes (and related neuropathy)	ICD-10: E10-E14, O24.0-O24.4, O24.9<br>ICD-9: 250, 648A, 648W
	Essential tremors	The ASM primidone is used for the treatment of essential tremors along with a READ code in CPRD or ICD-10 code (any diagnostic field) in HES for essential tremors, anytime prior to pregnancy start.		

Other covariate definitions

The following covariates were adjusted for in all analyses.

Covariate	CPRD (UK) Definition	Type	DOHaD (Sweden) Definition	Type
**Maternal age**	Maternal age at the start of each pregnancy as defined by the Pregnancy Register. We adjusted for age and age cubed to account for non-linear trends.	Continuous + cubic term	Same (per RTB)	Continuous + cubic term
**Geographical region**	The geographical region of the mother's general practice was identified using the CPRD practice file.	Categorical (East of England, East Midlands, London, North East, North West, South Central, South East Coast, South West, West Midlands Yorkshire and Humber, Northern Ireland, Scotland, Wales)	Maternal residential region	Categorical (6 "super regions" which govern local health care: 1=Northern Sweden, 2=Middle Sweden, 3=Stockholm, 4=Southeast Sweden, 5=West Sweden, 6=Southern Sweden)
**Socioeconomic position**	Household deprivation quintile (Index of Multiple Deprivation (IMD)). This is a regional based deprivation score. Patient-level IMD quintiles were obtained through linkage with deprivation data for 76.97% of patients living in England. Practice-level IMD data were used for the 23.03% of patients in England where patient-level data were unavailable. Patient-level IMD data are not available for Wales, Scotland or Northern Ireland so practice-level IMD data were used for all patients living in these countries. Patient level IMD was therefore used for 52.78% of all patients and practice level IMD was used for 47.22%.	Quintiles – entered as continuous variable	Maternal education level (primary, secondary, university)	Categorical
			Disposable income at birth, family weighted	Quintiles
**Evidence of alcohol problems or illicit drug use**	We used a binary measure to indicate alcohol problems or illicit drug use.<br><br>Evidence of alcohol problems was identified through medical codes indicating high alcohol consumption or prescriptions for the treatment of severe alcohol use. Records relating to alcohol	Binary	Diagnosis of Mental and behavioural disorders due to psychoactive substance use, before pregnancy (ever)	Binary

Covariate	CPRD (UK) Definition	Type	DOHaD (Sweden) Definition	Type
	consumption were identified from the maternal clinical file and additional clinical details file in CPRD and prescriptions for the treatment of severe alcohol use were identified in the therapy files. Evidence of hazardous drinking was defined as the consumption of ≥43 units/week, by the presence of a Read code indicating heavy drinking or by a relevant prescription. We first looked for information on alcohol consumption during each pregnancy. If there were no records during pregnancy, the most recent record in the 5 years prior was identified and used.<br><br>Women with pre-pregnancy illicit drug use were identified in one of three ways: 1) by searching for relevant Read codes in the mother's medical records up to 3 years before the estimated start of pregnancy; 2) by searching for relevant Read codes in the mother's additional clinical details records up to 3 years before the estimated LMP; and 3) by searching the mother's prescription records for drugs used in the treatment of illicit drug use.			
**Gravidity at pregnancy start**	Gravidity was taken from the pregnancy register using the order of pregnancies to the same mother.	Categorical (1,2,3,4,5+)	Parity	Categorical
**Health care utilization in the year before pregnancy**	Health care utilization in the year before pregnancy was assessed using the number of GP consultations involving an interaction with a healthcare professional.	Categorical (0-3, 4-10 10+)	Number of inpatient or specialized outpatient visits	Categorical
**Number of incident seizures in the year before pregnancy**	Incident seizures were identified by a Read code in the clinical or referral CPRD files, an ICD-10 code in the first diagnostic position in the HES APC linked dataset, or a Read code, ICD-10 code	Binary (0 vs 1 or more)	Inpatient or specialized outpatient visits with recorded epilepsy,	Binary (0 vs 1 or more)

Covariate	CPRD (UK) Definition	Type	DOHaD (Sweden) Definition	Type
	or an A&E diagnosis of an epilepsy related central nervous system condition in the HES A&E linked dataset. Incidents had to occur on separate days to be counted.		status epilepticus or convulsion	
**Medications taken during the periconceptional period**	Medication use in the 365 days prior to pregnancy start was ascertained from the therapy file. We created separate variables for antidepressant use and antipsychotic use.	Two binary variables (no antidepressant/ antipsychotic use vs antidepressant/ antipsychotic use)	Antidepressant (N06) and antipsychotic use (N05), during pregnancy. Two separate variables.	Binary x2
**Vomiting or prescription of antiemetics during pregnancy**	Presence of a read code for vomiting in the clinical or referral files or a prescription for an antiemetic in the therapy files during the pregnancy period.	Binary	A04, during pregnancy.	Binary
**Calendar year of start of pregnancy**	Taken as the year of the estimated start date of pregnancy from the pregnancy register.	Categorical (3 year groupings)	Taken as the year of the estimated start date of pregnancy from the pregnancy register.	Categorical (3 year groupings)
**Maternal neurodevelopmental condition diagnosis before pregnancy start**	A maternal diagnosis of autism, ID or ADHD in CPRD (using Read codes), HES admitted patient care or HES outpatient care (using ICD-10 codes) that occurred before pregnancy start.	Binary (no NDD, any NDD)		

Missing data

CPRD GOLD (UK)

We excluded 3 children who had missing sex information. Details on how missing prescription information were dealt with are provided in the section titled “Prescription data cleaning procedure – CPRD (UK)” above. Following the data cleaning procedure for prescriptions, the exposure, outcome and all covariates selected for adjustment in models were complete.

DOHaD (Sweden)

Pregnancies where information was missing on maternal residential region (N=4,608), household education (N=5,400), or maternal country of birth (N=203), and 2 pregnancies with infeasible dates were excluded from the cohort. In total 10,213 individuals (0.4% of the cohort) were excluded for missing data in any of these covariates.

Statistical analysis

Follow-up time

Follow-up time for children differed slightly by cohort due to the nature of the datasets. In CPRD end of data collection for the individual child constituted the age at transfer out of the practice (i.e. the age at which the child moved and the GP practice no longer collected data on them) or age at last collection date for the practice (i.e. the age of the child at the time the practice stopped providing data to CPRD). In DOHaD this constituted the age at migration of the child out of Sweden. The end of follow-up for the two cohorts also differed. For CPRD the end of follow-up was August 01 2021. For DOHaD the end of follow-up was December 31, 2021.

Supplementary Results

Supplementary Tables

Table S1 – Country and ASM specific exposure counts and follow up time

	CPRD (UK)			DOHaD (Sweden)		
ASM	Number exposed in pregnancy	Mean follow up in years (SD)	Median follow up in years (IQR)	Number exposed in pregnancy	Mean follow up in years (SD)	Median follow up in years (IQR)
No ASM	514066	7.97 (4.45)	7.52 (4.10-11.55)	2651210	13.04 (7.5)	12.51 (6.5-19.29)
Carbamazepine	603	8.97 (4.49)	9.17 (5.31-12.54)	2427	15.24 (6.93)	15.8 (9.52-21.08)
Gabapentin	564	5.74 (3.61)	4.93 (3.11-7.46)	864	6.93 (5.14)	5.24 (3.05-9.34)
Lamotrigine	939	6.98 (4.140)	6.18 (3.53-9.94)	5035	7.57 (4.95)	6.48 (3.61-10.66)
Levetiracetam	193	5.03 (3.330)	4.19 (2.56-6.79)	613	5.43 (3.71)	4.41 (2.49-7.56)
Phenytoin	46	10.57 (4.38)	10.75 (7.82-13.62)	194	19.66 (6.00)	21.1 (17.37-24.4)
Pregabalin	408	5.45 (3.00)	4.92 (3.07-7.25)	1307	7.62 (3.69)	7.70 (4.56-10.71)
Topiramate	154	6.16 (3.39)	5.41 (3.59-8.40)	264	8.38 (4.68)	8.23 (4.21-12.07)
Valproate	423	9.18 (4.50)	9.32 (5.54-12.83)	1178	14.58 (6.44)	14.88 (9.8-19.72)
Other	78	7.39 (4.25)	6.83 (3.89-10.67)	465	12.08 (7.01)	11.12 (6.24-17.45)
Polytherapy	573	7.56 (4.43)	7.07 (3.81-11.13)	1167	9.67 (5.86)	8.72 (4.99-13.17)
Total cohort	518047	7.96 (4.45)	7.51 (4.10-11.55)	2664724	13.03 (7.49)	12.49 (6.49-19.27)

Table S2 – Primary Analysis: Country specific adjusted marginal risk results

Outcome	ASM	CPRD (UK)				DOHaD (Sweden)			
		Number with outcome [a]	Risk as % (95% CI)			Number with outcome	Risk as % (95% CI)		
			Age 4	Age 8	Age 12		Age 4	Age 8	Age 12
Autism	No ASM	7657	0.46 (0.44-0.48)	1.87 (1.80-1.94)	3.95 (3.80-4.11)	68788	0.25 (0.24-0.26)	1.18 (1.15-1.22)	2.67 (2.60-2.74)
	Carbamazepine	21	0.71 (0.43-1.16)	2.87 (1.78-4.63)	6.00 (3.77-9.54)	136	0.30 (0.25-0.36)	1.43 (1.19-1.71)	3.21 (2.69-3.83)
	Gabapentin	16	0.65 (0.40-1.06)	2.65 (1.66-4.25)	5.56 (3.52-8.80)	21	0.24 (0.16-0.37)	1.15 (0.76-1.76)	2.61 (1.72-3.95)
	Lamotrigine	20	0.50 (0.31-0.82)	2.05 (1.27-3.30)	4.33 (2.71-6.90)	134	0.21 (0.17-0.25)	0.99 (0.82-1.18)	2.23 (1.87-2.65)
	Levetiracetam	<5	0.41 (0.10-1.68)	1.68 (0.42-6.73)	3.56 (0.91-13.91)	8	0.20 (0.10-0.40)	0.96 (0.48-1.91)	2.16 (1.09-4.28)
	Phenytoin	<5	0.55 (0.08-3.66)	2.23 (0.34-14.44)	4.69 (0.76-29.08)	10	0.24 (0.13-0.45)	1.15 (0.62-2.14)	2.61 (1.42-4.78)
	Pregabalin	7	0.36 (0.17-0.77)	1.49 (0.71-3.12)	3.16 (1.53-6.54)	47	0.18 (0.13-0.23)	0.83 (0.63-1.11)	1.89 (1.42-2.51)
	Topiramate	<5	0.14 (0.02-1.02)	0.58 (0.08-4.16)	1.24 (0.18-8.80)	13	0.31 (0.18-0.54)	1.49 (0.87-2.55)	3.35 (1.98-5.66)
	Valproate	14	0.69 (0.40-1.20)	2.80 (1.63-4.81)	5.87 (3.47-9.91)	111	0.45 (0.37-0.55)	2.13 (1.75-2.58)	4.73 (3.93-5.71)
	Other	<5	0.75 (0.24-2.33)	3.03 (1.00-9.18)	6.32 (2.16-18.52)	30	0.32 (0.22-0.46)	1.50 (1.06-2.15)	3.38 (2.39-4.78)
	Polytherapy	19	0.71 (0.43-1.17)	2.88 (1.77-4.67)	6.02 (3.76-9.63)	73	0.37 (0.29-0.48)	1.77 (1.40-2.24)	3.96 (3.15-4.99)
Intellectual disability	No ASM	1119	0.06 (0.06-0.07)	0.20 (0.19-0.22)	0.44 (0.40-0.48)	24323	0.16 (0.15-0.16)	0.53 (0.52-0.54)	0.96 (0.94-0.98)
	Carbamazepine	<5	0.11 (0.04-0.31)	0.34 (0.12-0.98)	0.73 (0.26-2.11)	64	0.20 (0.15-0.26)	0.69 (0.53-0.90)	1.23 (0.94-1.60)
	Gabapentin	0	0.00 (0.00-0.00)	0.00 (0.00-0.00)	0.00 (0.00-0.00)	7	0.16 (0.08-0.34)	0.55 (0.26-1.14)	0.98 (0.47-2.05)
	Lamotrigine	<5	0.10 (0.04-0.28)	0.32 (0.11-0.89)	0.69 (0.25-1.91)	60	0.17 (0.13-0.23)	0.58 (0.45-0.77)	1.05 (0.80-1.37)
	Levetiracetam	0	0.00 (0.00-0.00)	0.00 (0.00-0.00)	0.00 (0.00-0.00)	<5	0.10 (0.03-0.32)	0.35 (0.11-1.10)	0.63 (0.20-1.96)
	Phenytoin	0	0.00 (0.00-0.00)	0.00 (0.00-0.00)	0.00 (0.00-0.00)	<5	0.11 (0.03-0.33)	0.36 (0.12-1.12)	0.65 (0.21-2.01)
	Pregabalin	0	0.00 (0.00-0.00)	0.00 (0.00-0.00)	0.00 (0.00-0.00)	16	0.13 (0.08-0.22)	0.46 (0.28-0.75)	0.82 (0.50-1.34)
	Topiramate	<5	0.20 (0.03-1.45)	0.63 (0.09-4.55)	1.36 (0.19-9.72)	7	0.38 (0.18-0.79)	1.28 (0.61-2.67)	2.28 (1.10-4.73)
	Valproate	<5	0.07 (0.02-0.28)	0.21 (0.05-0.88)	0.46 (0.11-1.90)	64	0.41 (0.32-0.54)	1.40 (1.08-1.82)	2.49 (1.92-3.23)
	Other	0	0.00 (0.00-0.00)	0.00 (0.00-0.00)	0.00 (0.00-0.00)	8	0.14 (0.07-0.29)	0.48 (0.24-0.97)	0.87 (0.43-1.73)
	Polytherapy	<5	0.12 (0.04-0.35)	0.39 (0.14-1.10)	0.85 (0.31-2.36)	43	0.32 (0.23-0.44)	1.08 (0.79-1.48)	1.93 (1.41-2.65)
Attention Deficit Hyperactivity Disorder	No ASM	4794	0.08 (0.07-0.09)	0.93 (0.88-0.97)	2.10 (1.99-2.21)	147608	0.04 (0.03-0.05)	1.47 (1.29-1.67)	5.70 (5.09-6.38)
	Carbamazepine	13	0.12 (0.07-0.21)	1.39 (0.81-2.40)	3.14 (1.84-5.34)	257	0.04 (0.03-0.05)	1.53 (1.28-1.84)	5.94 (5.04-7.01)
	Gabapentin	9	0.14 (0.07-0.28)	1.63 (0.85-3.12)	3.67 (1.95-6.91)	39	0.04 (0.03-0.06)	1.65 (1.18-2.29)	6.35 (4.67-8.64)
	Lamotrigine	14	0.13 (0.07-0.23)	1.51 (0.87-2.64)	3.40 (1.97-5.87)	243	0.03 (0.03-0.04)	1.27 (1.05-1.52)	4.96 (4.19-5.87)
	Levetiracetam	<5	0.12 (0.02-0.63)	1.37 (0.27-7.05)	3.09 (0.62-15.39)	14	0.04 (0.02-0.07)	1.49 (0.87-2.53)	5.78 (3.51-9.52)
	Phenytoin	<5	0.17 (0.02-1.23)	1.97 (0.29-13.53)	4.41 (0.68-28.69)	26	0.04 (0.03-0.07)	1.66 (1.11-2.47)	6.39 (4.41-9.27)
	Pregabalin	<5	0.07 (0.02-0.24)	0.86 (0.27-2.71)	1.94 (0.62-6.06)	88	0.03 (0.02-0.03)	0.99 (0.77-1.26)	3.91 (3.10-4.92)
	Topiramate	<5	0.06 (0.01-0.43)	0.67 (0.09-4.89)	1.52 (0.21-10.93)	15	0.03 (0.02-0.05)	1.18 (0.71-1.98)	4.65 (2.86-7.57)
	Valproate	10	0.12 (0.06-0.25)	1.37 (0.67-2.81)	3.09 (1.52-6.25)	164	0.05 (0.04-0.06)	1.73 (1.41-2.11)	6.65 (5.55-7.98)
	Other	<5	0.08 (0.01-0.62)	0.97 (0.13-7.05)	2.21 (0.31-15.56)	48	0.04 (0.03-0.05)	1.39 (1.02-1.89)	5.40 (4.06-7.20)
	Polytherapy	8	0.09 (0.04-0.20)	1.04 (0.48-2.25)	2.37 (1.11-5.05)	102	0.04 (0.03-0.06)	1.64 (1.29-2.07)	6.32 (5.10-7.83)

[a] Number with outcome at the end of follow up

Table S3 – **Primary Analysis:** Pooled adjusted marginal risk results

Outcome	ASM	Risk as % (95% CI)			Weight for DOHaD (Sweden) (%)		
		Age 4	Age 8	Age 12	Age 4	Age 8	Age 12
Autism	No ASM	0.29 (0.28-0.30)	1.39 (1.36-1.42)	3.01 (2.94-3.08)	75.3	64.6	69.5
	Carbamazepine	0.33 (0.28-0.40)	1.56 (1.31-1.84)	3.47 (2.94-4.09)	87.8	87.6	87.4
	Gabapentin	0.38 (0.27-0.52)	1.67 (1.22-2.29)	3.67 (2.70-5.00)	55.8	55.3	54.8
	Lamotrigine	0.23 (0.19-0.27)	1.08 (0.91-1.27)	2.42 (2.05-2.85)	88	87.8	87.7
	Levetiracetam	0.23 (0.12-0.43)	1.07 (0.58-1.98)	2.39 (1.30-4.40)	80.2	80.1	80
	Phenytoin	0.26 (0.15-0.48)	1.23 (0.69-2.21)	2.76 (1.55-4.91)	90.3	90.2	90.1
	Pregabalin	0.19 (0.15-0.25)	0.90 (0.69-1.17)	2.02 (1.55-2.63)	87	87	86.9
	Topiramate	0.30 (0.18-0.50)	1.40 (0.83-2.34)	3.13 (1.89-5.20)	93	93.1	93.3
	Valproate	0.47 (0.39-0.57)	2.19 (1.83-2.63)	4.85 (4.07-5.78)	88.8	88.8	88.7
	Other	0.34 (0.24-0.48)	1.61 (1.15-2.25)	3.59 (2.58-4.99)	90.9	90.7	90.5
	Polytherapy	0.42 (0.34-0.53)	1.94 (1.57-2.40)	4.29 (3.49-5.28)	81	80.8	80.7
Intellectual disability	No ASM	0.15 (0.14-0.15)	0.50 (0.49-0.51)	0.92 (0.90-0.94)	92.7	93.9	95.1
	Carbamazepine	0.19 (0.15-0.25)	0.66 (0.51-0.85)	1.19 (0.92-1.54)	94.1	94.1	94.1
	Gabapentin	0.16 (0.08-0.34)	0.55 (0.26-1.14)	0.98 (0.47-2.05)	100	100	100
	Lamotrigine	0.17 (0.13-0.22)	0.56 (0.43-0.73)	1.02 (0.79-1.32)	93.4	93.5	93.5
	Levetiracetam	0.10 (0.03-0.32)	0.35 (0.11-1.10)	0.63 (0.20-1.96)	100	100	100
	Phenytoin	0.11 (0.03-0.33)	0.36 (0.12-1.12)	0.65 (0.21-2.01)	100	100	100
	Pregabalin	0.13 (0.08-0.22)	0.46 (0.28-0.75)	0.82 (0.50-1.34)	100	100	100
	Topiramate	0.35 (0.17-0.70)	1.17 (0.59-2.33)	2.14 (1.08-4.24)	87.7	87.8	87.9
	Valproate	0.39 (0.30-0.50)	1.31 (1.02-1.70)	2.36 (1.83-3.05)	96.7	96.8	96.8
	Other	0.14 (0.07-0.29)	0.48 (0.24-0.97)	0.87 (0.43-1.73)	100	100	100
	Polytherapy	0.29 (0.22-0.40)	0.99 (0.73-1.34)	1.80 (1.33-2.43)	91.1	91.2	91.3
Attention Deficit Hyperactivity Disorder	No ASM	0.06 (0.06-0.07)	0.98 (0.94-1.03)	2.50 (2.39-2.63)	**31.2**	**13.3**	**17.6**
	Carbamazepine	0.05 (0.04-0.06)	1.52 (1.28-1.81)	5.62 (4.80-6.58)	89.4	89.9	91.3
	Gabapentin	0.06 (0.04-0.08)	1.64 (1.22-2.21)	5.72 (4.34-7.54)	79	79.2	80.9
	Lamotrigine	0.04 (0.03-0.05)	1.29 (1.08-1.54)	4.80 (4.09-5.64)	89.7	90.1	91.3
	Levetiracetam	0.04 (0.03-0.07)	1.48 (0.89-2.45)	5.47 (3.40-8.81)	90.3	90.5	91.2
	Phenytoin	0.05 (0.03-0.07)	1.67 (1.13-2.47)	6.30 (4.38-9.07)	95.8	95.9	96.2
	Pregabalin	0.03 (0.02-0.04)	0.98 (0.77-1.25)	3.80 (3.03-4.77)	95.5	95.6	96
	Topiramate	0.03 (0.02-0.05)	1.14 (0.69-1.88)	4.36 (2.72-7.00)	93.6	93.8	94.3
	Valproate	0.05 (0.04-0.06)	1.70 (1.40-2.06)	6.34 (5.32-7.56)	92.3	92.7	93.8
	Other	0.04 (0.03-0.05)	1.37 (1.01-1.86)	5.30 (3.99-7.04)	97.5	97.6	97.9
	Polytherapy	0.05 (0.04-0.06)	1.57 (1.26-1.97)	5.88 (4.78-7.22)	91.1	91.5	92.6

Table S4 – Primary Analysis: Country specific risk difference results

Outcome	ASM	CPRD (UK) - Risk Difference as % (95% CI)			DOHaD (Sweden) - Risk Difference as % (95% CI)		
		Age 4	Age 8	Age 12	Age 4	Age 8	Age 12
Autism	No ASM	0 (Reference)	0 (Reference)	0 (Reference)	0 (Reference)	0 (Reference)	0 (Reference)
	Carbamazepine	0.25 (-0.10, 0.60)	1.00 (-0.38, 2.37)	2.05 (-0.74, 4.83)	0.05 (-0.00, 0.11)	0.24 (-0.01, 0.50)	0.54 (-0.02, 1.10)
	Gabapentin	0.20 (-0.12, 0.51)	0.78 (-0.46, 2.03)	1.61 (-0.93, 4.16)	-0.01 (-0.11, 0.10)	-0.03 (-0.52, 0.46)	-0.07 (-1.15, 1.02)
	Lamotrigine	0.05 (-0.20, 0.29)	0.18 (-0.80, 1.16)	0.38 (-1.64, 2.40)	-0.04 (-0.08, -0.01)	-0.20 (-0.37, -0.03)	-0.44 (-0.83, -0.06)
	Levetiracetam	-0.05 (-0.62, 0.53)	-0.19 (-2.52, 2.14)	-0.39 (-5.25, 4.46)	-0.05 (-0.19, 0.09)	-0.23 (-0.89, 0.43)	-0.51 (-1.98, 0.97)
	Phenytoin	0.09 (-0.95, 1.13)	0.36 (-3.80, 4.52)	0.74 (-7.82, 9.29)	-0.01 (-0.16, 0.15)	-0.03 (-0.74, 0.68)	-0.07 (-1.64, 1.51)
	Pregabalin	-0.09 (-0.37, 0.18)	-0.38 (-1.48, 0.72)	-0.79 (-3.09, 1.51)	-0.07 (-0.12, -0.02)	-0.35 (-0.59, -0.11)	-0.78 (-1.31, -0.25)
	Topiramate	-0.32 (-0.60, -0.04)	-1.29 (-2.43, -0.14)	-2.71 (-5.14, -0.27)	0.07 (-0.11, 0.24)	0.31 (-0.49, 1.10)	0.68 (-1.08, 2.43)
	Valproate	0.23 (-0.15, 0.62)	0.94 (-0.58, 2.45)	1.92 (-1.16, 4.99)	0.20 (0.11, 0.29)	0.94 (0.54, 1.35)	2.06 (1.18, 2.94)
	Other	0.29 (-0.56, 1.14)	1.16 (-2.20, 4.52)	2.37 (-4.42, 9.16)	0.07 (-0.05, 0.18)	0.32 (-0.21, 0.85)	0.71 (-0.46, 1.88)
	Polytherapy	0.25 (-0.10, 0.60)	1.01 (-0.38, 2.40)	2.07 (-0.76, 4.89)	0.13 (0.04, 0.21)	0.59 (0.17, 1.00)	1.29 (0.38, 2.20)
Intellectual disability	No ASM	0 (Reference)	0 (Reference)	0 (Reference)	0 (Reference)	0 (Reference)	0 (Reference)
	Carbamazepine	0.04 (-0.07, 0.16)	0.14 (-0.22, 0.50)	0.30 (-0.48, 1.07)	0.05 (-0.01, 0.10)	0.15 (-0.03, 0.34)	0.27 (-0.05, 0.60)
	Gabapentin	-	-	-	0.00 (-0.12, 0.12)	0.01 (-0.39, 0.42)	0.02 (-0.70, 0.75)
	Lamotrigine	0.04 (-0.07, 0.14)	0.12 (-0.21, 0.44)	0.25 (-0.45, 0.95)	0.02 (-0.03, 0.06)	0.05 (-0.11, 0.21)	0.09 (-0.19, 0.37)
	Levetiracetam	-	-	-	-0.05 (-0.17, 0.06)	-0.18 (-0.58, 0.22)	-0.33 (-1.04, 0.39)
	Phenytoin	-	-	-	-0.05 (-0.17, 0.07)	-0.17 (-0.58, 0.24)	-0.31 (-1.04, 0.42)
	Pregabalin	-	-	-	-0.02 (-0.09, 0.04)	-0.07 (-0.30, 0.15)	-0.13 (-0.54, 0.27)
	Topiramate	0.14 (-0.26, 0.53)	0.43 (-0.82, 1.67)	0.92 (-1.75, 3.60)	0.22 (-0.06, 0.50)	0.74 (-0.20, 1.68)	1.32 (-0.34, 2.98)
	Valproate	0.00 (-0.09, 0.10)	0.01 (-0.29, 0.31)	0.02 (-0.63, 0.67)	0.26 (0.15, 0.37)	0.87 (0.50, 1.23)	1.54 (0.89, 2.19)
	Other	-	-	-	-0.01 (-0.11, 0.08)	-0.05 (-0.39, 0.29)	-0.09 (-0.69, 0.51)
	Polytherapy	0.06 (-0.07, 0.19)	0.19 (-0.21, 0.59)	0.41 (-0.45, 1.28)	0.16 (0.06, 0.26)	0.55 (0.20, 0.89)	0.98 (0.37, 1.58)
Attention Deficit Hyperactivity Disorder	No ASM	0 (Reference)	0 (Reference)	0 (Reference)	0 (Reference)	0 (Reference)	0 (Reference)
	Carbamazepine	0.04 (-0.03, 0.11)	0.47 (-0.29, 1.22)	1.04 (-0.63, 2.70)	0.00 (-0.00, 0.01)	0.07 (-0.13, 0.26)	0.24 (-0.48, 0.96)
	Gabapentin	0.06 (-0.03, 0.16)	0.71 (-0.35, 1.77)	1.57 (-0.75, 3.89)	0.00 (-0.01, 0.02)	0.18 (-0.33, 0.68)	0.65 (-1.18, 2.48)
	Lamotrigine	0.05 (-0.02, 0.13)	0.59 (-0.25, 1.43)	1.30 (-0.55, 3.15)	-0.01 (-0.01, -0.00)	-0.20 (-0.37, -0.03)	-0.74 (-1.36, -0.12)
	Levetiracetam	0.04 (-0.16, 0.24)	0.44 (-1.80, 2.69)	0.99 (-3.97, 5.95)	0.00 (-0.02, 0.02)	0.02 (-0.75, 0.79)	0.08 (-2.73, 2.89)
	Phenytoin	0.09 (-0.25, 0.43)	1.05 (-2.75, 4.84)	2.31 (-5.95, 10.56)	0.01 (-0.01, 0.02)	0.19 (-0.44, 0.81)	0.69 (-1.58, 2.96)
	Pregabalin	-0.01 (-0.09, 0.08)	-0.07 (-1.05, 0.91)	-0.16 (-2.36, 2.05)	-0.01 (-0.02, -0.01)	-0.48 (-0.69, -0.27)	-1.79 (-2.59, -1.00)
	Topiramate	-0.02 (-0.14, 0.09)	-0.26 (-1.59, 1.07)	-0.58 (-3.58, 2.41)	-0.01 (-0.02, 0.01)	-0.28 (-0.87, 0.30)	-1.05 (-3.26, 1.15)
	Valproate	0.04 (-0.05, 0.13)	0.44 (-0.54, 1.43)	0.99 (-1.19, 3.16)	0.01 (-0.00, 0.01)	0.26 (-0.01, 0.53)	0.95 (-0.02, 1.92)
	Other	0.00 (-0.16, 0.17)	0.05 (-1.88, 1.98)	0.11 (-4.20, 4.42)	-0.00 (-0.01, 0.01)	-0.08 (-0.47, 0.30)	-0.30 (-1.72, 1.13)
	Polytherapy	0.01 (-0.06, 0.08)	0.12 (-0.68, 0.92)	0.27 (-1.53, 2.06)	0.00 (-0.00, 0.01)	0.17 (-0.15, 0.49)	0.62 (-0.55, 1.78)

Table S5 – Primary Analysis: Pooled risk difference (relative to no ASM) results

Outcome	ASM	Risk Difference as % (95% CI)			Weight for DOHaD (Sweden) (%)		
		Age 4	Age 8	Age 12	Age 4	Age 8	Age 12
Autism	No ASM	0 (Reference)	0 (Reference)	0 (Reference)	-	-	-
	Carbamazepine	0.06 (0.00, 0.11)	0.27 (0.02, 0.52)	0.60 (0.05, 1.15)	97.6	96.7	96.1
	Gabapentin	0.01 (-0.08, 0.11)	0.08 (-0.38, 0.53)	0.19 (-0.81, 1.19)	90.1	86.8	84.7
	Lamotrigine	-0.04 (-0.08, -0.00)	-0.19 (-0.36, -0.02)	-0.41 (-0.79, -0.03)	97.8	96.9	96.4
	Levetiracetam	-0.05 (-0.18, 0.09)	-0.23 (-0.86, 0.41)	-0.50 (-1.91, 0.91)	94.5	92.6	91.6
	Phenytoin	-0.00 (-0.15, 0.15)	-0.02 (-0.72, 0.68)	-0.04 (-1.59, 1.51)	97.9	97.2	96.7
	Pregabalin	-0.08 (-0.12, -0.03)	-0.35 (-0.58, -0.12)	-0.78 (-1.30, -0.26)	96.7	95.5	94.9
	Topiramate	-0.04 (-0.18, 0.11)	-0.22 (-0.87, 0.44)	-0.48 (-1.91, 0.95)	72.7	67.3	65.8
	Valproate	0.20 (0.12, 0.29)	0.94 (0.55, 1.33)	2.05 (1.21, 2.90)	94.9	93.3	92.5
	Other	0.07 (-0.04, 0.19)	0.34 (-0.19, 0.87)	0.76 (-0.40, 1.91)	98.2	97.5	97.1
	Polytherapy	0.13 (0.05, 0.22)	0.62 (0.22, 1.02)	1.36 (0.50, 2.23)	93.9	91.8	90.6
Intellectual disability	No ASM	0 (Reference)	0 (Reference)	0 (Reference)	-	-	-
	Carbamazepine	0.04 (-0.00, 0.09)	0.15 (-0.01, 0.31)	0.28 (-0.03, 0.58)	81.7	79.5	85
	Gabapentin	0.00 (-0.12, 0.12)	0.01 (-0.39, 0.42)	0.02 (-0.70, 0.75)	100	100	100
	Lamotrigine	0.02 (-0.02, 0.06)	0.06 (-0.08, 0.21)	0.11 (-0.15, 0.38)	83	80.9	86.1
	Levetiracetam	-0.05 (-0.17, 0.06)	-0.18 (-0.58, 0.22)	-0.33 (-1.04, 0.39)	100	100	100
	Phenytoin	-0.05 (-0.17, 0.07)	-0.17 (-0.58, 0.24)	-0.31 (-1.04, 0.42)	100	100	100
	Pregabalin	-0.02 (-0.09, 0.04)	-0.07 (-0.30, 0.15)	-0.13 (-0.54, 0.27)	100	100	100
	Topiramate	0.19 (-0.04, 0.42)	0.63 (-0.12, 1.38)	1.21 (-0.20, 2.62)	66.7	63.8	72.1
	Valproate	0.11 (0.04, 0.18)	0.35 (0.12, 0.59)	0.78 (0.32, 1.24)	43.1	40.3	50.3
	Other	-0.01 (-0.11, 0.08)	-0.05 (-0.39, 0.29)	-0.09 (-0.69, 0.51)	100	100	100
	Polytherapy	0.12 (0.04, 0.20)	0.40 (0.14, 0.66)	0.79 (0.29, 1.29)	61	58	67
Attention Deficit Hyperactivity Disorder	No ASM	0 (Reference)	0 (Reference)	0 (Reference)	-	-	-
	Carbamazepine	0.00 (-0.00, 0.01)	0.09 (-0.10, 0.28)	0.37 (-0.29, 1.03)	99.3	93.6	84.3
	Gabapentin	0.01 (-0.01, 0.02)	0.28 (-0.18, 0.73)	1.00 (-0.43, 2.44)	97.9	81.5	61.7
	Lamotrigine	-0.01 (-0.01, -0.00)	-0.17 (-0.33, -0.01)	-0.53 (-1.12, 0.05)	99.6	96.2	90
	Levetiracetam	0.00 (-0.02, 0.02)	0.06 (-0.66, 0.79)	0.30 (-2.15, 2.74)	98.9	89.6	75.7
	Phenytoin	0.01 (-0.01, 0.02)	0.21 (-0.41, 0.83)	0.80 (-1.39, 2.99)	99.7	97.4	93
	Pregabalin	-0.01 (-0.02, -0.01)	-0.46 (-0.67, -0.25)	-1.61 (-2.35, -0.86)	99.5	95.5	88.5
	Topiramate	-0.01 (-0.02, 0.01)	-0.28 (-0.82, 0.26)	-0.89 (-2.66, 0.89)	98.1	83.5	64.8
	Valproate	0.01 (-0.00, 0.01)	0.27 (0.01, 0.53)	0.96 (0.07, 1.84)	99.3	93	83.4
	Other	-0.00 (-0.01, 0.01)	-0.08 (-0.45, 0.30)	-0.26 (-1.61, 1.10)	99.6	96.2	90.2
	Polytherapy	0.00 (-0.00, 0.01)	0.16 (-0.14, 0.46)	0.51 (-0.46, 1.49)	98.5	86.3	70.4

Table S6 – **Primary Analysis:** Country specific and combined hazard ratio results

		CPRD (UK)		DOHaD (Sweden)		Combined
Outcome	ASM	Weighting	Adjusted HR (95 % CI)	Weighting	Adjusted HR (95 % CI)	Adjusted HR (95 % CI)
Autism	No ASM	-	1 (Reference)	-	1 (Reference)	1 (Reference)
	Carbamazepine	12	1.55 (0.94-2.53)	88	1.21 (1.01-1.45)	1.25 (1.05-1.48)
	Gabapentin	44	1.43 (0.88-2.32)	56	0.97 (0.64-1.50)	1.15 (0.84-1.59)
	Lamotrigine	11.9	1.10 (0.67-1.78)	88.1	0.83 (0.69-0.99)	0.86 (0.72-1.01)
	Levetiracetam	19.7	0.90 (0.22-3.67)	80.3	0.80 (0.40-1.62)	0.82 (0.44-1.54)
	Phenytoin	9.7	1.19 (0.18-8.05)	90.3	0.97 (0.52-1.82)	0.99 (0.55-1.80)
	Pregabalin	12.9	0.79 (0.37-1.67)	87.1	0.70 (0.53-0.93)	0.71 (0.54-0.93)
	Topiramate	7.1	0.31 (0.04-2.21)	92.9	1.26 (0.73-2.18)	1.14 (0.68-1.93)
	Valproate	11	1.51 (0.87-2.63)	89	1.82 (1.49-2.21)	1.78 (1.48-2.14)
	Other	9	1.63 (0.52-5.12)	91	1.28 (0.89-1.83)	1.30 (0.93-1.84)
	Polytherapy	18.8	1.55 (0.94-2.55)	81.2	1.51 (1.19-1.91)	1.51 (1.22-1.88)
Intellectual disability	No ASM	-	1 (Reference)	-	1 (Reference)	1 (Reference)
	Carbamazepine	6	1.67 (0.58-4.85)	94	1.28 (0.98-1.68)	1.30 (1.01-1.69)
	Gabapentin	-	-	100	1.02 (0.49-2.15)	1.02 (0.49-2.15)
	Lamotrigine	6.6	1.57 (0.57-4.38)	93.4	1.09 (0.83-1.44)	1.12 (0.86-1.46)
	Levetiracetam	-	-	100	0.66 (0.21-2.05)	0.66 (0.21-2.05)
	Phenytoin	-	-	100	0.67 (0.22-2.10)	0.67 (0.22-2.10)
	Pregabalin	-	-	100	0.86 (0.52-1.40)	0.86 (0.52-1.40)
	Topiramate	12.3	3.12 (0.43-22.82)	87.7	2.40 (1.14-5.06)	2.48 (1.23-4.98)
	Valproate	3.3	1.04 (0.25-4.34)	96.7	2.64 (2.02-3.44)	2.56 (1.97-3.32)
	Other	-	-	100	0.90 (0.45-1.81)	0.90 (0.45-1.81)
	Polytherapy	8.9	1.95 (0.70-5.43)	91.1	2.03 (1.47-2.80)	2.02 (1.49-2.75)
Attention Deficit Hyperactivity Disorder	No ASM		1 (Reference)		1 (Reference)	1 (Reference)
	Carbamazepine	5.4	1.51 (0.87-2.61)	94.6	1.05 (0.92-1.19)	1.07 (0.94-1.21)
	Gabapentin	18.5	1.77 (0.92-3.43)	81.5	1.13 (0.82-1.54)	1.22 (0.92-1.63)
	Lamotrigine	5.2	1.64 (0.93-2.89)	94.8	0.86 (0.75-0.98)	0.89 (0.78-1.01)
	Levetiracetam	9.1	1.48 (0.28-7.83)	90.9	1.02 (0.60-1.72)	1.05 (0.64-1.74)
	Phenytoin	3.7	2.15 (0.30-15.39)	96.3	1.13 (0.77-1.67)	1.16 (0.79-1.70)
	Pregabalin	3.2	0.92 (0.29-2.93)	96.8	0.67 (0.54-0.82)	0.67 (0.55-0.83)
	Topiramate	6	0.71 (0.10-5.32)	94	0.80 (0.48-1.33)	0.80 (0.49-1.30)
	Valproate	4.5	1.48 (0.71-3.07)	95.5	1.18 (1.01-1.39)	1.20 (1.02-1.40)
	Other	2	1.05 (0.14-7.77)	98	0.94 (0.71-1.26)	0.95 (0.71-1.25)
	Polytherapy	6.2	1.13 (0.52-2.45)	93.8	1.12 (0.92-1.37)	1.12 (0.92-1.36)

Table S7 – Heterogeneity Tests: Wald tests of hazard ratio comparisons

Outcome	ASM	Wald test of heterogeneity									
		CPRD versus DOHaD primary analysis results		Primary analysis versus sibling comparison results		Primary analysis versus indication restricted results					
						Primary analysis vs epilepsy		Primary analysis vs psychiatric		Primary analysis vs somatic	
		Wald statistic [a]	P value [b]	Wald statistic [a]	P value [b]	Wald statistic [a]	P value [b]	Wald statistic [a]	P value [b]	Wald statistic [a]	P value [b]
Autism	No ASM	-	-	-	-	-	-	-	-	-	-
	Carbamazepine	0.842	0.35889	0.000	0.98563	0.036	0.84883	0.257	0.61188	0.521	0.47037
	Gabapentin	1.343	0.24652	1.361	0.24344	0.036	0.85008	0.073	0.78720	0.131	0.71748
	Lamotrigine	1.124	0.28914	0.005	0.94264	0.003	0.95974	1.664	0.19701	0.014	0.90470
	Levetiracetam	0.017	0.89497	0.369	0.54349	0.086	0.76906	0.298	0.58492	-	-
	Phenytoin	0.039	0.84441	15.696	**0.00007**	0.120	0.72924	1.189	0.27550	0.050	0.82298
	Pregabalin	0.089	0.76597	0.644	0.42230	-	-	0.630	0.42738	0.245	0.62082
	Topiramate	1.832	0.17588	0.092	0.76209	2.214	0.13676	0.125	0.72396	0.036	0.84875
	Valproate	0.378	0.53874	3.821	**0.05062**	0.817	0.36602	0.401	0.52662	0.288	0.59159
	Other	0.163	0.68614	0.007	0.93123	0.234	0.62887	0.108	0.74265	0.251	0.61633
	Polytherapy	0.011	0.91465	0.269	0.60414	1.868	0.17172	0.057	0.81054	0.821	0.36477
Intellectual disability	No ASM	-	-	-	-	-	-	-	-	-	-
	Carbamazepine	0.225	0.63563	1.100	0.29420	0.305	0.58061	2.019	0.15533	1.190	0.27535
	Gabapentin	-	-	0.672	0.41245	-	-	0.000	0.98327	0.010	0.91945
	Lamotrigine	0.454	0.50050	0.025	0.87393	0.260	0.60982	0.194	0.65999	1.494	0.22163
	Levetiracetam	-	-	0.004	0.95148	0.141	0.70702	-	-	-	-
	Phenytoin	-	-	0.025	0.87448	0.010	0.92024	-	-	0.364	0.54628
	Pregabalin	-	-	0.033	0.85621	-	-	0.275	0.60007	0.001	0.97895
	Topiramate	0.058	0.80965	0.499	0.48001	2.063	0.15092	0.266	0.60627	0.076	0.78241
	Valproate	1.582	0.20850	1.704	0.19181	0.101	0.75028	2.171	0.14064	1.008	0.31534
	Other	-	-	0.248	0.61842	0.099	0.75258	0.395	0.52956	0.004	0.94727
	Polytherapy	0.006	0.93956	2.410	0.12055	1.339	0.24715	1.688	0.19380	0.001	0.97810
Attention Deficit Hyperactivity Disorder	No ASM	-	-	-	-	-	-	-	-	-	-
	Carbamazepine	1.577	0.20916	0.254	0.61428	0.046	0.82931	0.010	0.92007	0.292	0.58872
	Gabapentin	1.485	0.22294	0.066	0.79761	0.356	0.55049	0.010	0.91917	1.242	0.26511
	Lamotrigine	4.739	**0.02949**	0.081	0.77621	0.036	0.85039	1.383	0.23967	0.114	0.73516
	Levetiracetam	0.179	0.67182	0.391	0.53165	0.017	0.89512	0.204	0.65123	5.920	**0.01497**
	Phenytoin	0.389	0.53298	15.681	**0.00007**	0.004	0.94752	0.282	0.59527	0.363	0.54701
	Pregabalin	0.285	0.59315	4.503	**0.03383**	28.684	**<0.00001**	0.213	0.64435	2.345	0.12570
	Topiramate	0.013	0.91091	1.100	0.29433	0.177	0.67413	0.002	0.96610	0.149	0.69976
	Valproate	0.345	0.55691	0.997	0.31793	0.027	0.87008	0.453	0.50081	4.670	**0.03070**

	Other	0.010	0.91906	5.810	**0.01593**	0.386	0.53424	0.243	0.62178	0.667	0.41407
	Polytherapy	0.000	0.99018	3.128	**0.07698**	0.032	0.85903	0.089	0.76549	0.323	0.56966

[a] – Wald statistic calculated on the log hazard ratio scale as $\frac{(\log(HR_1)-\log(HR_2))^2}{V_1+V_2}$ where V_i is the square of the standard error for $\log(HR_i)$. Note that this assumes that there is no covariance between HR_1 and HR_2 which would inflate the Wald statistic value if covariance in estimates were to be positive (as would be expected). P-values are therefore conservative.

[b] – Wald statistic compared to the χ^2 distribution with 1 degree of freedom. By default, the p-value is a 2-sided test.

Table S8 – Discordant Sibling Analysis: Country specific and combined results

Outcome	ASM	CPRD (UK)		DOHaD (Sweden)		Combined
		Weighting	Adjusted HR (95 % CI)	Weighting	Adjusted HR (95 % CI)	Adjusted HR (95 % CI)
Autism	No ASM	-	1 (Reference)	-	1 (Reference)	1 (Reference)
	Carbamazepine	21.5	1.79 (0.66-4.79)	78.5	1.13 (0.68-1.90)	1.25 (0.79-1.98)
	Gabapentin	52.6	0.97 (0.41-2.25)	47.4	0.59 (0.24-1.43)	0.76 (0.41-1.41)
	Lamotrigine	22	1.21 (0.55-2.68)	78	0.76 (0.50-1.16)	0.84 (0.58-1.23)
	Levetiracetam	50.9	0.59 (0.12-3.01)	49.1	0.50 (0.10-2.62)	0.55 (0.17-1.74)
	Phenytoin	73	15.33 (4.45-52.85)	27	5.34 (0.70-40.88)	11.53 (4.00-33.20)
	Pregabalin	37.9	0.54 (0.23-1.28)	62.1	1.24 (0.63-2.42)	0.91 (0.54-1.54)
	Topiramate	19.6	0.31 (0.03-3.01)	80.4	1.95 (0.64-5.98)	1.36 (0.50-3.72)
	Valproate	22.3	0.81 (0.28-2.31)	77.7	1.13 (0.65-1.99)	1.05 (0.64-1.72)
	Other	36.2	1.64 (0.42-6.39)	63.8	1.08 (0.39-3.01)	1.25 (0.55-2.85)
	Polytherapy	32.1	1.12 (0.43-2.93)	67.9	1.39 (0.72-2.70)	1.30 (0.75-2.24)
Intellectual disability	No ASM	-	1 (Reference)	-	1 (Reference)	1 (Reference)
	Carbamazepine	37.7	8.21 (3.14-21.44)	62.3	0.74 (0.35-1.57)	1.84 (1.02-3.32)
	Gabapentin	-	-	100	1.91 (0.52-7.03)	1.91 (0.52-7.03)
	Lamotrigine	15	2.42 (0.46-12.72)	85	1.04 (0.52-2.10)	1.19 (0.62-2.25)
	Levetiracetam	-	-	100	0.71 (0.08-6.47)	0.71 (0.08-6.47)
	Phenytoin	-	-	100	0.53 (0.03-8.50)	0.53 (0.03-8.50)
	Pregabalin	-	-	100	0.77 (0.26-2.26)	0.77 (0.26-2.26)
	Topiramate	33.8	1.35 (0.14-12.70)	66.2	1.51 (0.30-7.50)	1.45 (0.40-5.36)
	Valproate	18.5	0.30 (0.06-1.59)	81.5	2.23 (1.01-4.92)	1.54 (0.76-3.14)
	Other	-	-	100	0.57 (0.11-3.00)	0.57 (0.11-3.00)
	Polytherapy	11.3	1.24 (0.10-15.12)	88.7	0.97 (0.40-2.36)	1.00 (0.43-2.31)
Attention Deficit Hyperactivity Disorder	No ASM	-	1 (Reference)	-	1 (Reference)	1 (Reference)
	Carbamazepine	8.1	0.86 (0.26-2.91)	91.9	0.98 (0.68-1.41)	0.97 (0.69-1.37)
	Gabapentin	17.3	3.44 (0.96-12.35)	82.7	0.90 (0.50-1.61)	1.13 (0.67-1.92)
	Lamotrigine	5.4	5.97 (1.69-21.11)	94.6	0.76 (0.56-1.03)	0.85 (0.63-1.14)
	Levetiracetam	27.5	4.06 (0.52-31.68)	72.5	1.06 (0.30-3.77)	1.54 (0.52-4.51)
	Phenytoin	45.3	38.49 (9.14-162.11)	54.7	2.98 (0.81-11.01)	9.48 (3.60-24.95)
	Pregabalin	7.5	1.62 (0.38-7.03)	92.5	1.06 (0.70-1.62)	1.10 (0.74-1.64)
	Topiramate	14.3	0.25 (0.02-2.67)	85.7	0.51 (0.19-1.35)	0.46 (0.19-1.13)
	Valproate	15	4.00 (1.39-11.47)	85	1.26 (0.81-1.96)	1.49 (0.99-2.25)
	Other	18.6	8.83 (1.69-46.00)	81.4	1.81 (0.82-3.98)	2.43 (1.19-4.95)
	Polytherapy	21.7	4.04 (1.51-10.85)	78.3	1.39 (0.83-2.35)	1.76 (1.11-2.78)

Table S9 – Active Comparator Analysis: Country specific and combined results

Outcome	ASM	CPRD (UK)		DOHaD (Sweden)		Combined
		Weighting	Adjusted HR (95 % CI)	Weighting	Adjusted HR (95 % CI)	Adjusted HR (95 % CI)
Autism	Lamotrigine	-	1 (Reference)	-	1 (Reference)	1 (Reference)
	No ASM	11.9	0.91 (0.56-1.48)	88.1	1.21 (1.01-1.44)	1.17 (0.99-1.38)
	Carbamazepine	12.0	1.41 (0.73-2.71)	88.0	1.46 (1.14-1.86)	1.45 (1.16-1.82)
	Gabapentin	31.3	1.30 (0.66-2.58)	68.7	1.18 (0.74-1.87)	1.21 (0.83-1.78)
	Levetiracetam	20.5	0.82 (0.20-3.34)	79.5	0.97 (0.47-1.99)	0.94 (0.50-1.77)
	Phenytoin	9.8	1.09 (0.15-7.66)	90.2	1.18 (0.62-2.24)	1.17 (0.63-2.15)
	Pregabalin	12.5	0.72 (0.30-1.76)	87.5	0.85 (0.60-1.18)	0.83 (0.61-1.13)
	Topiramate	7.2	0.28 (0.04-2.15)	92.8	1.52 (0.86-2.69)	1.35 (0.78-2.33)
	Valproate	11.9	1.38 (0.69-2.74)	88.1	2.19 (1.70-2.82)	2.07 (1.63-2.63)
	Other	9.6	1.49 (0.44-5.03)	90.4	1.54 (1.04-2.29)	1.53 (1.05-2.24)
	Polytherapy	16.7	1.42 (0.75-2.68)	83.3	1.82 (1.36-2.42)	1.74 (1.34-2.26)
Intellectual disability	Lamotrigine	-	1 (Reference)	-	1 (Reference)	1 (Reference)
	No ASM	6.6	0.64 (0.23-1.77)	93.4	0.91 (0.70-1.20)	0.89 (0.69-1.16)
	Carbamazepine	6.4	1.06 (0.27-4.16)	93.6	1.17 (0.82-1.68)	1.17 (0.83-1.65)
	Gabapentin	-	-	100	0.93 (0.43-2.05)	0.93 (0.43-2.05)
	Levetiracetam	-	-	100	0.60 (0.19-1.92)	0.60 (0.19-1.92)
	Phenytoin	-	-	100	0.61 (0.19-1.96)	0.61 (0.19-1.96)
	Pregabalin	-	-	100	0.78 (0.45-1.37)	0.78 (0.45-1.37)
	Topiramate	11.7	1.98 (0.23-17.02)	88.3	2.19 (1.00-4.81)	2.17 (1.04-4.53)
	Valproate	4.3	0.66 (0.12-3.51)	95.7	2.41 (1.69-3.44)	2.28 (1.61-3.22)
	Other	-	-	100	0.83 (0.39-1.73)	0.83 (0.39-1.73)
	Polytherapy	7.9	1.24 (0.32-4.74)	92.1	1.86 (1.25-2.75)	1.80 (1.23-2.62)
Attention Deficit Hyperactivity Disorder	Lamotrigine	-	1 (Reference)	-	1 (Reference)	1 (Reference)
	Carbamazepine	5.2	0.61 (0.35-1.07)	94.8	1.16 (1.02-1.33)	1.12 (0.99-1.28)
	Gabapentin	5.2	0.92 (0.43-1.96)	94.8	1.22 (1.02-1.45)	1.20 (1.01-1.42)
	No ASM	13.4	1.08 (0.46-2.56)	86.6	1.31 (0.93-1.84)	1.27 (0.93-1.75)
	Levetiracetam	8.9	0.90 (0.16-5.10)	91.1	1.18 (0.69-2.02)	1.15 (0.69-1.93)
	Phenytoin	3.9	1.31 (0.17-9.91)	96.1	1.32 (0.88-1.97)	1.32 (0.88-1.96)
	Pregabalin	3.5	0.56 (0.15-2.06)	96.5	0.77 (0.61-0.99)	0.77 (0.60-0.98)
	Topiramate	6.0	0.43 (0.06-3.43)	94.0	0.93 (0.55-1.57)	0.89 (0.54-1.48)
	Valproate	5.0	0.90 (0.38-2.15)	95.0	1.38 (1.13-1.68)	1.35 (1.11-1.63)
	Other	2.2	0.64 (0.08-5.00)	97.8	1.10 (0.80-1.50)	1.08 (0.80-1.47)
	Polytherapy	6.0	0.69 (0.27-1.72)	94.0	1.30 (1.03-1.64)	1.25 (1.00-1.57)

Table S10 – Indication Stratified Analysis: Counts of exposed and exposed with each outcome in each country according to indication

Outcome	ASM	CPRD (UK)						DOHaD (Sweden)					
		Epilepsy		Psychiatric		Somatic		Epilepsy		Psychiatric		Somatic	
		N exposed	N exposed with outcome [a]	N exposed	N exposed with outcome [a]	N exposed	N exposed with outcome [a]	N exposed	N exposed with outcome [a]	N exposed	N exposed with outcome [a]	N exposed	N exposed with outcome [a]
Autism	No ASM	4075	78	183,004	3701	69,091	1195	10,769	425	189,904	6058	244,909	5582
	Carbamazepine	460	17	290	11	132	5	1842	98	317	29	335	24
	Gabapentin	18	<5	499	15	279	11	43	<5	359	7	487	9
	Lamotrigine	791	17	469	11	180	7	2383	53	2832	84	1171	32
	Levetiracetam	174	<5	100	0	27	0	556	8	124	<5	147	0
	Phenytoin	45	<5	18	<5	7	0	147	7	12	0	16	<5
	Pregabalin	9	0	380	6	192	<5	45	0	928	34	594	20
	Topiramate	43	<5	115	0	108	0	71	6	149	6	119	<5
	Valproate	354	11	218	11	90	6	792	82	275	26	157	13
	Other	34	0	61	<5	23	<5	225	14	165	15	115	9
	Polytherapy	505	16	315	14	150	7	912	63	419	28	294	16
Intellectual disability	No ASM	4075	13	183,004	447	69,091	165	10,769	194	189,904	1912	244,909	1182
	Carbamazepine	460	<5	290	<5	132	0	1842	56	317	7	335	13
	Gabapentin	18	0	499	0	279	0	43	0	359	<5	487	<5
	Lamotrigine	791	<5	469	<5	180	<5	2383	34	2832	31	1171	17
	Levetiracetam	174	0	100	0	27	0	556	<5	124	0	147	0
	Phenytoin	45	0	18	0	7	0	147	<5	12	0	16	<5
	Pregabalin	9	0	380	0	192	0	45	0	928	13	594	7
	Topiramate	43	0	115	<5	108	<5	71	5	149	<5	119	<5
	Valproate	354	<5	218	<5	90	0	792	51	275	9	157	6
	Other	34	0	61	0	23	0	225	5	165	<5	115	<5
	Polytherapy	505	<5	315	<5	150	<5	912	40	419	8	294	9
Attention Deficit Hyperactivity Disorder	No ASM	4075	38	183,004	2385	69,091	797	10,769	929	189,904	11,585	244,909	11,466
	Carbamazepine	460	11	290	11	132	<5	1842	190	317	39	335	33
	Gabapentin	18	0	499	9	279	7	43	5	359	12	487	22
	Lamotrigine	791	14	469	6	180	<5	2383	109	2832	140	1171	48
	Levetiracetam	174	<5	100	<5	27	<5	556	14	124	<5	147	<5
	Phenytoin	45	<5	18	<5	7	0	147	23	12	<5	16	<5
	Pregabalin	9	<5	380	<5	192	<5	45	0	928	58	594	43
	Topiramate	43	0	115	<5	108	<5	71	<5	149	8	119	7

Outcome	ASM	CPRD (UK)						DOHaD (Sweden)					
		Epilepsy		Psychiatric		Somatic		Epilepsy		Psychiatric		Somatic	
		N exposed	N exposed with outcome [a]	N exposed	N exposed with outcome [a]	N exposed	N exposed with outcome [a]	N exposed	N exposed with outcome [a]	N exposed	N exposed with outcome [a]	N exposed	N exposed with outcome [a]
	Valproate	354	8	218	6	90	<5	792	113	275	39	157	14
	Other	34	0	61	<5	23	0	225	24	165	22	115	15
	Polytherapy	505	7	315	5	150	<5	912	78	419	42	294	24

[a] Number with outcome at the end of follow up

Table S11 – Indication Stratified Analysis: Country specific adjusted marginal risk results

		CPRD (UK)			DOHaD (Sweden)		
		Risk as % (95% CI) at age 12 stratified by indication			Risk as % (95% CI) at age 12 stratified by indication		
Outcome	ASM	Epilepsy	Psychiatric	Somatic	Epilepsy	Psychiatric	Somatic
Autism	No ASM	5.93 (4.18-8.40)	5.99 (5.65-6.36)	6.08 (5.46-6.77)	4.92 (4.32-5.61)	6.39 (5.85-6.98)	5.11 (4.62-5.65)
	Carbamazepine	10.02 (5.87-17.10)	8.07 (4.51-14.42)	8.08 (2.92-22.39)	6.03 (4.84-7.52)	8.60 (5.98-12.37)	7.36 (4.88-11.09)
	Gabapentin	12.81 (1.55-105.59)	8.54 (5.37-13.58)	10.65 (6.30-18.00)	3.30 (0.50-21.69)	5.59 (2.74-11.40)	4.12 (2.18-7.76)
	Lamotrigine	6.82 (4.04-11.54)	6.84 (3.79-12.35)	8.13 (3.49-18.97)	4.48 (3.41-5.88)	6.45 (5.16-8.06)	4.21 (2.93-6.06)
	Levetiracetam	6.19 (1.51-25.46)	-	-	5.02 (2.60-9.71)	7.50 (2.55-22.06)	-
	Phenytoin	7.59 (1.26-45.72)	16.19 (3.22-81.27)	-	4.33 (2.11-8.91)	-	5.98 (1.58-22.63)
	Pregabalin	-	4.58 (2.11-9.98)	5.42 (2.11-13.95)	-	5.54 (3.97-7.75)	4.08 (2.64-6.31)
	Topiramate	4.49 (0.63-32.14)	-	-	13.31 (6.63-26.73)	6.17 (2.87-13.28)	5.22 (2.05-13.29)
	Valproate	8.73 (4.73-16.12)	11.16 (6.30-19.75)	12.98 (6.23-27.05)	10.34 (8.27-12.91)	9.07 (6.26-13.14)	6.55 (3.86-11.13)
	Other	-	8.00 (2.11-30.39)	12.08 (2.03-71.95)	7.82 (4.79-12.77)	9.08 (5.62-14.66)	7.54 (4.05-14.02)
	Polytherapy	9.14 (5.47-15.26)	10.39 (6.22-17.36)	8.27 (3.87-17.69)	9.62 (7.55-12.27)	9.32 (6.47-13.42)	5.81 (3.55-9.49)
Intellectual disability	No ASM	0.71 (0.34-1.47)	0.60 (0.51-0.70)	0.66 (0.50-0.88)	2.01 (1.70-2.38)	1.45 (1.33-1.58)	1.22 (1.11-1.34)
	Carbamazepine	1.41 (0.43-4.66)	0.42 (0.06-3.01)	-	3.43 (2.56-4.60)	1.11 (0.52-2.38)	2.27 (1.26-4.11)
	Gabapentin	-	-	-	-	1.51 (0.49-4.64)	1.33 (0.50-3.53)
	Lamotrigine	1.37 (0.42-4.50)	0.84 (0.20-3.55)	3.03 (0.77-11.94)	2.95 (2.08-4.17)	1.78 (1.23-2.56)	1.65 (0.98-2.77)
	Levetiracetam	-	-	-	2.24 (0.74-6.83)	-	-
	Phenytoin	-	-	-	1.84 (0.60-5.67)	-	1.67 (0.23-11.91)
	Pregabalin	-	-	-	-	1.52 (0.88-2.63)	1.07 (0.50-2.25)
	Topiramate	-	2.46 (0.35-17.07)	3.18 (0.47-21.56)	12.50 (5.72-27.29)	1.65 (0.42-6.51)	1.02 (0.15-7.12)
	Valproate	1.13 (0.25-5.11)	1.05 (0.26-4.27)	-	6.28 (4.69-8.40)	2.19 (1.13-4.24)	1.99 (0.88-4.51)
	Other	-	-	-	2.69 (1.14-6.37)	0.80 (0.20-3.21)	1.05 (0.26-4.21)
	Polytherapy	2.11 (0.73-6.09)	1.29 (0.43-3.87)	2.79 (0.83-9.46)	5.82 (4.23-8.00)	1.51 (0.74-3.08)	1.91 (0.96-3.81)
Attention Deficit Hyperactivity Disorder	No ASM	2.82 (1.84-4.32)	3.25 (3.00-3.51)	3.01 (2.64-3.44)	11.44 (10.45-12.53)	13.22 (10.73-16.27)	11.12 (8.48-14.59)
	Carbamazepine	6.85 (3.65-12.83)	5.85 (3.34-10.23)	3.21 (0.79-13.07)	11.28 (9.70-13.12)	11.88 (8.31-16.97)	10.70 (7.05-16.23)
	Gabapentin	-	5.91 (3.16-11.05)	7.65 (3.86-15.18)	16.83 (8.28-34.19)	12.53 (7.31-21.50)	14.39 (9.33-22.19)
	Lamotrigine	5.46 (2.89-10.32)	4.00 (1.80-8.90)	5.31 (1.69-16.70)	9.66 (8.07-11.56)	13.22 (10.25-17.05)	9.19 (6.25-13.53)
	Levetiracetam	4.16 (0.69-25.19)	6.66 (1.65-26.95)	19.24 (8.90-41.58)	11.16 (7.09-17.58)	13.94 (5.78-33.63)	6.62 (1.79-24.53)
	Phenytoin	9.62 (1.71-54.06)	11.69 (1.91-71.62)	-	12.56 (8.75-18.02)	13.90 (4.07-47.51)	9.12 (3.15-26.42)
	Pregabalin	48.15 (25.23-91.90)	2.20 (0.55-8.82)	1.99 (0.26-14.98)	-	10.01 (7.26-13.79)	10.16 (6.92-14.92)
	Topiramate	-	2.92 (0.41-20.94)	3.07 (0.43-21.96)	11.38 (4.91-26.38)	10.49 (5.43-20.29)	10.47 (5.17-21.20)
	Valproate	4.90 (2.08-11.52)	4.18 (1.68-10.40)	4.53 (1.09-18.88)	13.15 (11.02-15.68)	13.66 (9.68-19.28)	7.08 (4.01-12.51)
	Other	-	3.89 (0.56-27.08)	-	12.29 (8.67-17.42)	13.97 (9.20-21.21)	13.07 (7.85-21.76)
	Polytherapy	4.47 (1.92-10.40)	3.08 (1.10-8.59)	3.29 (0.74-14.56)	12.49 (10.21-15.29)	15.50 (11.12-21.62)	10.87 (6.90-17.13)

Table S12 – Indication Stratified Analysis: Pooled adjusted marginal risk at age 12 results

Outcome	ASM	Risk as % (95% CI) at age 12 stratified by indication			Weight for DOHaD (Sweden) (%)		
		Epilepsy	Psychiatric	Somatic	Epilepsy	Psychiatric	Somatic
Autism	No ASM	5.03 (4.45-5.69)	6.11 (5.82-6.42)	5.54 (5.15-5.96)	88.3	31	53.5
	Carbamazepine	6.49 (5.29-7.95)	8.45 (6.20-11.50)	7.46 (5.10-10.91)	85.7	71.8	86
	Gabapentin	6.02 (1.48-24.54)	7.53 (5.10-11.11)	7.23 (4.83-10.84)	55.6	29.7	40.7
	Lamotrigine	4.89 (3.84-6.23)	6.50 (5.27-8.01)	4.67 (3.34-6.51)	79	87.5	84.5
	Levetiracetam	5.21 (2.87-9.48)	7.50 (2.55-22.06)	-	82.2	100	-
	Phenytoin	4.68 (2.40-9.14)	16.19 (3.22-81.27)	5.98 (1.58-22.63)	86.2	0	100
	Pregabalin	-	5.38 (3.96-7.32)	4.29 (2.89-6.37)	-	84.3	82.4
	Topiramate	11.79 (6.11-22.76)	6.17 (2.87-13.28)	5.22 (2.05-13.29)	88.9	100	100
	Valproate	10.14 (8.22-12.50)	9.64 (7.07-13.16)	8.28 (5.39-12.73)	88.5	70.3	65.7
	Other	7.82 (4.79-12.77)	8.95 (5.70-14.05)	7.93 (4.41-14.25)	100	88.6	89.2
	Polytherapy	9.54 (7.65-11.88)	9.67 (7.18-13.01)	6.44 (4.27-9.74)	82	66.5	70.6
Intellectual disability	No ASM	1.91 (1.62-2.25)	1.19 (1.11-1.29)	1.15 (1.05-1.25)	95	77.9	90.3
	Carbamazepine	3.27 (2.46-4.34)	0.98 (0.48-1.99)	2.27 (1.26-4.11)	94.4	86.9	100
	Gabapentin	-	1.51 (0.49-4.64)	1.33 (0.50-3.53)	-	100	100
	Lamotrigine	2.77 (1.99-3.87)	1.70 (1.19-2.42)	1.78 (1.10-2.88)	92.1	93.9	87.6
	Levetiracetam	2.24 (0.74-6.83)	-	-	100	-	-
	Phenytoin	1.84 (0.60-5.67)	-	1.67 (0.23-11.91)	100	-	100
	Pregabalin	-	1.52 (0.88-2.63)	1.07 (0.50-2.25)	-	100	100
	Topiramate	12.50 (5.72-27.29)	1.88 (0.61-5.78)	1.82 (0.46-7.11)	100	66.6	49.1
	Valproate	5.90 (4.43-7.86)	1.92 (1.06-3.48)	1.99 (0.88-4.51)	96.4	81.9	100
	Other	2.69 (1.14-6.37)	0.80 (0.20-3.21)	1.05 (0.26-4.21)	100	100	100
	Polytherapy	5.35 (3.94-7.25)	1.44 (0.79-2.62)	2.10 (1.15-3.82)	91.7	70.4	75.8
Attention Deficit Hyperactivity Disorder	No ASM	10.77 (9.85-11.77)	3.87 (3.60-4.17)	3.87 (3.44-4.36)	95.7	12.5	19.2
	Carbamazepine	10.98 (9.48-12.72)	9.68 (7.16-13.08)	9.70 (6.51-14.47)	94.5	71.1	91.9
	Gabapentin	16.83 (8.28-34.19)	9.10 (6.04-13.69)	12.02 (8.33-17.33)	100	57.3	71.5
	Lamotrigine	9.26 (7.79-11.01)	11.84 (9.29-15.09)	8.69 (6.03-12.53)	92.7	90.8	89.8
	Levetiracetam	10.52 (6.77-16.34)	11.30 (5.36-23.81)	14.62 (7.52-28.41)	94	71.6	25.7
	Phenytoin	12.42 (8.72-17.68)	13.16 (4.76-36.40)	9.12 (3.15-26.42)	95.8	68.5	100
	Pregabalin	48.15 (25.23-91.90)	9.27 (6.78-12.67)	9.60 (6.58-14.00)	0	94.9	96.5
	Topiramate	11.38 (4.91-26.38)	9.23 (4.94-17.24)	9.10 (4.69-17.69)	100	89.9	88.6
	Valproate	12.63 (10.63-15.01)	11.79 (8.54-16.27)	6.66 (3.93-11.30)	95.9	87.5	86.3
	Other	12.29 (8.67-17.42)	13.20 (8.78-19.86)	13.07 (7.85-21.76)	100	95.6	100
	Polytherapy	11.82 (9.71-14.38)	13.30 (9.69-18.24)	9.82 (6.36-15.16)	94.6	90.5	91.5

Table S13 – Sensitivity Analysis: Pooled hazard ratio results

NDD	ASM	Hazard Ratio (95% CI)				Weighting for Sweden			
			Sensitivity analyses				Sensitivity analyses		
		Primary analysis	Minimum of 4 years follow up	Trimester 1 exposure	2 prescriptions for exposure definition	Primary analysis	Minimum of 4 years follow up	Trimester 1 exposure	2 prescriptions for exposure definition
ASD	No ASM	1 (Reference)	1 (Reference)	1 (Reference)	1 (Reference)	-	-	-	-
	Carbamazepine	1.25 (1.05-1.48)	1.26 (1.06-1.49)	1.36 (1.04-1.78)	1.28 (0.97-1.67)	88.0%	88.0%	72.0%	73.4%
	Gabapentin	1.15 (0.84-1.59)	1.20 (0.87-1.66)	1.16 (0.80-1.68)	1.08 (0.67-1.73)	56.0%	56.0%	45.6%	45.9%
	Lamotrigine	0.86 (0.72-1.01)	0.87 (0.74-1.03)	0.90 (0.75-1.09)	0.85 (0.70-1.03)	88.1%	88.0%	85.7%	83.7%
	Levetiracetam	0.82 (0.44-1.54)	0.88 (0.47-1.65)	1.19 (0.65-2.17)	1.00 (0.54-1.88)	80.3%	80.4%	82.0%	80.4%
	Phenytoin	0.99 (0.55-1.80)	1.00 (0.55-1.81)	1.19 (0.18-8.05)	1.25 (0.19-8.45)	90.3%	90.3%	.%	.%
	Pregabalin	0.71 (0.54-0.93)	0.69 (0.52-0.91)	0.79 (0.60-1.04)	0.73 (0.52-1.02)	87.1%	92.1%	86.3%	83.4%
	Topiramate	1.14 (0.68-1.93)	1.07 (0.62-1.84)	1.38 (0.81-2.33)	1.17 (0.58-2.34)	92.9%	92.4%	92.9%	87.7%
	Valproate	1.78 (1.48-2.14)	1.79 (1.49-2.15)	1.65 (1.24-2.21)	1.71 (1.27-2.30)	89.0%	89.0%	71.2%	75.2%
	Other	1.30 (0.93-1.84)	1.31 (0.93-1.85)	1.37 (0.86-2.18)	1.22 (0.75-2.00)	91.0%	91.0%	83.5%	93.5%
	Polytherapy	1.51 (1.22-1.88)	1.50 (1.20-1.86)	1.51 (1.15-1.98)	1.47 (1.19-1.83)	81.2%	81.6%	73.0%	81.4%
ID	No ASM	1 (Reference)	1 (Reference)	1 (Reference)	1 (Reference)	-	-	-	-
	Carbamazepine	1.30 (1.01-1.69)	1.30 (1.00-1.69)	1.36 (0.87-2.13)	1.26 (0.83-1.92)	94.0%	94.0%	87.0%	88.7%
	Gabapentin	1.02 (0.49-2.15)	0.76 (0.32-1.83)	1.10 (0.46-2.64)	0.67 (0.17-2.69)	100.0%	100.0%	100.0%	100.0%
	Lamotrigine	1.12 (0.86-1.46)	1.15 (0.88-1.49)	1.11 (0.81-1.52)	1.01 (0.73-1.38)	93.4%	93.4%	90.7%	90.6%
	Levetiracetam	0.66 (0.21-2.05)	0.70 (0.22-2.18)	1.20 (0.44-3.25)	0.75 (0.24-2.35)	100.0%	100.0%	100.0%	100.0%
	Phenytoin	0.67 (0.22-2.10)	0.67 (0.21-2.08)	2.82 (0.39-20.20)	1.81 (0.25-12.91)	100.0%	100.0%	100.0%	100.0%
	Pregabalin	0.86 (0.52-1.40)	0.87 (0.53-1.42)	0.90 (0.53-1.53)	0.59 (0.28-1.24)	100.0%	100.0%	100.0%	100.0%
	Topiramate	2.48 (1.23-4.98)	2.52 (1.25-5.06)	2.47 (1.17-5.21)	3.85 (1.83-8.12)	87.7%	87.7%	86.0%	100.0%
	Valproate	2.56 (1.97-3.32)	2.51 (1.93-3.26)	2.09 (1.31-3.34)	2.45 (1.62-3.71)	96.7%	96.6%	94.6%	91.8%
	Other	0.90 (0.45-1.81)	0.90 (0.45-1.81)	0.98 (0.37-2.64)	0.86 (0.32-2.30)	100.0%	100.0%	100.0%	100.0%
	Polytherapy	2.02 (1.49-2.75)	2.04 (1.50-2.77)	2.17 (1.45-3.24)	1.88 (1.39-2.55)	91.1%	91.1%	85.0%	91.3%
ADHD	No ASM	1 (Reference)	1 (Reference)	1 (Reference)	1 (Reference)	-	-	-	-
	Carbamazepine	1.07 (0.94-1.21)	1.07 (0.94-1.21)	0.97 (0.77-1.24)	1.00 (0.79-1.27)	94.6%	94.6%	82.7%	82.9%
	Gabapentin	1.22 (0.92-1.63)	1.23 (0.93-1.63)	1.27 (0.89-1.82)	0.79 (0.47-1.34)	81.5%	81.5%	70.4%	77.8%
	Lamotrigine	0.89 (0.78-1.01)	0.89 (0.78-1.01)	0.85 (0.73-1.00)	0.90 (0.77-1.05)	94.8%	94.8%	93.5%	92.8%
	Levetiracetam	1.05 (0.64-1.74)	1.06 (0.64-1.75)	1.04 (0.58-1.87)	1.17 (0.67-2.04)	90.9%	90.8%	87.5%	88.4%
	Phenytoin	1.16 (0.79-1.70)	1.16 (0.79-1.70)	2.75 (1.14-6.62)	1.86 (0.77-4.48)	96.3%	96.3%	80.0%	80.1%
	Pregabalin	0.67 (0.55-0.83)	0.67 (0.54-0.82)	0.74 (0.60-0.91)	0.66 (0.50-0.86)	96.8%	97.8%	96.6%	96.3%
	Topiramate	0.80 (0.49-1.30)	0.80 (0.49-1.31)	0.96 (0.58-1.56)	0.80 (0.40-1.60)	94.0%	94.0%	94.0%	100.0%
	Valproate	1.20 (1.02-1.40)	1.20 (1.02-1.40)	1.19 (0.92-1.54)	1.26 (0.97-1.65)	95.5%	95.5%	88.8%	89.8%
	Other	0.95 (0.71-1.25)	0.95 (0.71-1.26)	0.98 (0.66-1.46)	0.89 (0.59-1.35)	98.0%	98.0%	96.1%	100.0%
	Polytherapy	1.12 (0.92-1.36)	1.12 (0.92-1.36)	1.09 (0.84-1.42)	1.11 (0.92-1.35)	93.8%	93.8%	90.0%	93.8%

Table S14 – Sensitivity Analysis: Comparison of primary analysis models including and excluding vomiting or antiemetics as a covariate

		CPRD (UK)		DOHaD (Sweden)		Pooled	
Outcome	ASM	Primary	Sensitivity *	Primary	Sensitivity *	Primary	Sensitivity *
Autism	No ASM	1 (Reference)	1 (Reference)	1 (Reference)	1 (Reference)	1 (Reference)	1 (Reference)
	Carbamazepine	1.55 (0.94-2.53)	1.53 (0.94-2.51)	1.21 (1.01-1.45)	1.21 (1.01-1.45)	1.25 (1.05-1.48)	1.24 (1.05-1.48)
	Gabapentin	1.43 (0.88-2.32)	1.47 (0.91-2.39)	0.97 (0.64-1.50)	0.98 (0.64-1.50)	1.15 (0.84-1.59)	1.17 (0.85-1.61)
	Lamotrigine	1.10 (0.67-1.78)	1.09 (0.67-1.78)	0.83 (0.69-0.99)	0.83 (0.69-0.99)	0.86 (0.72-1.01)	0.86 (0.72-1.01)
	Levetiracetam	0.90 (0.22-3.67)	0.87 (0.21-3.58)	0.80 (0.40-1.62)	0.80 (0.40-1.62)	0.82 (0.44-1.54)	0.82 (0.44-1.53)
	Phenytoin	1.19 (0.18-8.05)	1.19 (0.18-8.01)	0.97 (0.52-1.82)	0.98 (0.52-1.82)	0.99 (0.55-1.80)	0.99 (0.55-1.80)
	Pregabalin	0.79 (0.37-1.67)	0.81 (0.38-1.71)	0.70 (0.53-0.93)	0.70 (0.53-0.94)	0.71 (0.54-0.93)	0.71 (0.55-0.94)
	Topiramate	0.31 (0.04-2.21)	0.31 (0.04-2.26)	1.26 (0.73-2.18)	1.28 (0.74-2.21)	1.14 (0.68-1.93)	1.16 (0.68-1.96)
	Valproate	1.51 (0.87-2.63)	1.50 (0.86-2.61)	1.82 (1.49-2.21)	1.82 (1.49-2.21)	1.78 (1.48-2.14)	1.78 (1.48-2.14)
	Other	1.63 (0.52-5.12)	1.59 (0.50-4.99)	1.28 (0.89-1.83)	1.28 (0.89-1.83)	1.30 (0.93-1.84)	1.30 (0.92-1.84)
	Polytherapy	1.55 (0.94-2.55)	1.55 (0.94-2.56)	1.51 (1.19-1.91)	1.51 (1.19-1.92)	1.51 (1.22-1.88)	1.52 (1.22-1.88)
Intellectual disability	No ASM	1 (Reference)	1 (Reference)	1 (Reference)	1 (Reference)	1 (Reference)	1 (Reference)
	Carbamazepine	1.67 (0.58-4.85)	1.67 (0.58-4.84)	1.28 (0.98-1.68)	1.28 (0.98-1.68)	1.30 (1.01-1.69)	1.30 (1.01-1.69)
	Gabapentin	-	-	1.02 (0.49-2.15)	1.02 (0.49-2.15)	1.02 (0.49-2.15)	1.02 (0.49-2.15)
	Lamotrigine	1.57 (0.57-4.38)	1.56 (0.56-4.35)	1.09 (0.83-1.44)	1.09 (0.83-1.44)	1.12 (0.86-1.46)	1.12 (0.86-1.46)
	Levetiracetam	-	-	0.66 (0.21-2.05)	0.66 (0.21-2.05)	0.66 (0.21-2.05)	0.66 (0.21-2.05)
	Phenytoin	-	-	0.67 (0.22-2.10)	0.67 (0.22-2.10)	0.67 (0.22-2.10)	0.67 (0.22-2.10)
	Pregabalin	-	-	0.86 (0.52-1.40)	0.86 (0.52-1.40)	0.86 (0.52-1.40)	0.86 (0.52-1.40)
	Topiramate	3.12 (0.43-22.82)	3.18 (0.43-23.49)	2.40 (1.14-5.06)	2.41 (1.14-5.07)	2.48 (1.23-4.98)	2.49 (1.24-5.00)
	Valproate	1.04 (0.25-4.34)	1.03 (0.25-4.31)	2.64 (2.02-3.44)	2.64 (2.02-3.44)	2.56 (1.97-3.32)	2.56 (1.97-3.32)
	Other	-	-	0.90 (0.45-1.81)	0.90 (0.45-1.81)	0.90 (0.45-1.81)	0.90 (0.45-1.81)
	Polytherapy	1.95 (0.70-5.43)	1.96 (0.70-5.47)	2.03 (1.47-2.80)	2.03 (1.48-2.80)	2.02 (1.49-2.75)	2.03 (1.49-2.75)
Attention Deficit Hyperactivity Disorder	No ASM	1 (Reference)	1 (Reference)	1 (Reference)	1 (Reference)	1 (Reference)	1 (Reference)
	Carbamazepine	1.51 (0.87-2.61)	1.50 (0.86-2.60)	1.05 (0.92-1.19)	1.05 (0.92-1.19)	1.07 (0.94-1.21)	1.07 (0.94-1.21)
	Gabapentin	1.77 (0.92-3.43)	1.85 (0.95-3.58)	1.13 (0.82-1.54)	1.13 (0.83-1.55)	1.22 (0.92-1.63)	1.24 (0.93-1.64)
	Lamotrigine	1.64 (0.93-2.89)	1.63 (0.92-2.87)	0.86 (0.75-0.98)	0.86 (0.75-0.98)	0.89 (0.78-1.01)	0.89 (0.78-1.01)
	Levetiracetam	1.48 (0.28-7.83)	1.43 (0.27-7.47)	1.02 (0.60-1.72)	1.01 (0.60-1.72)	1.05 (0.64-1.74)	1.05 (0.63-1.73)
	Phenytoin	2.15 (0.30-15.39)	2.13 (0.30-15.27)	1.13 (0.77-1.67)	1.13 (0.77-1.67)	1.16 (0.79-1.70)	1.16 (0.79-1.70)
	Pregabalin	0.92 (0.29-2.93)	0.95 (0.30-3.02)	0.67 (0.54-0.82)	0.67 (0.54-0.82)	0.67 (0.55-0.83)	0.68 (0.55-0.83)
	Topiramate	0.71 (0.10-5.32)	0.72 (0.10-5.40)	0.80 (0.48-1.33)	0.81 (0.49-1.34)	0.80 (0.49-1.30)	0.80 (0.49-1.31)
	Valproate	1.48 (0.71-3.07)	1.46 (0.70-3.03)	1.18 (1.01-1.39)	1.19 (1.01-1.39)	1.20 (1.02-1.40)	1.20 (1.02-1.40)
	Other	1.05 (0.14-7.77)	1.01 (0.14-7.52)	0.94 (0.71-1.26)	0.94 (0.71-1.26)	0.95 (0.71-1.25)	0.95 (0.71-1.25)
	Polytherapy	1.13 (0.52-2.45)	1.12 (0.52-2.44)	1.12 (0.92-1.37)	1.12 (0.92-1.37)	1.12 (0.92-1.36)	1.12 (0.92-1.36)

* Sensitivity excluding vomiting or antiemetics as a covariate

Table S15 – Counts of antiseizure medications included in the polytherapy category

**ASM included in polytherapy**	**CPRD (UK), N (%) with polytherapy = 573 (100.00%)**	**DOHaD (Sweden), N (%) with polytherapy = 1167 (100.00%)**
Carbamazepine	204 (35.6%)	353 (30.2%)
Gabapentin	65 (11.3%)	92 (7.9%)
Lamotrigine	268 (46.8%)	794 (68.0%)
Levetiracetam	225 (39.3%)	449 (38.5%)
Phenytoin	27 (4.7%)	36 (3.1%)
Pregabalin	49 (8.6%)	187 (16.0%)
Topiramate	57 (10.0%)	157 (13.5%)
Valproate	145 (25.3%)	347 (29.7%)
Other	205 (35.8%)	0 (0.0%)
Note that rows within a column are not mutually exclusive and so the sum of percentages will be greater than 100%		

Supplementary Figures

Figure S1 – Flowchart of cohort derivation for CPRD (UK)

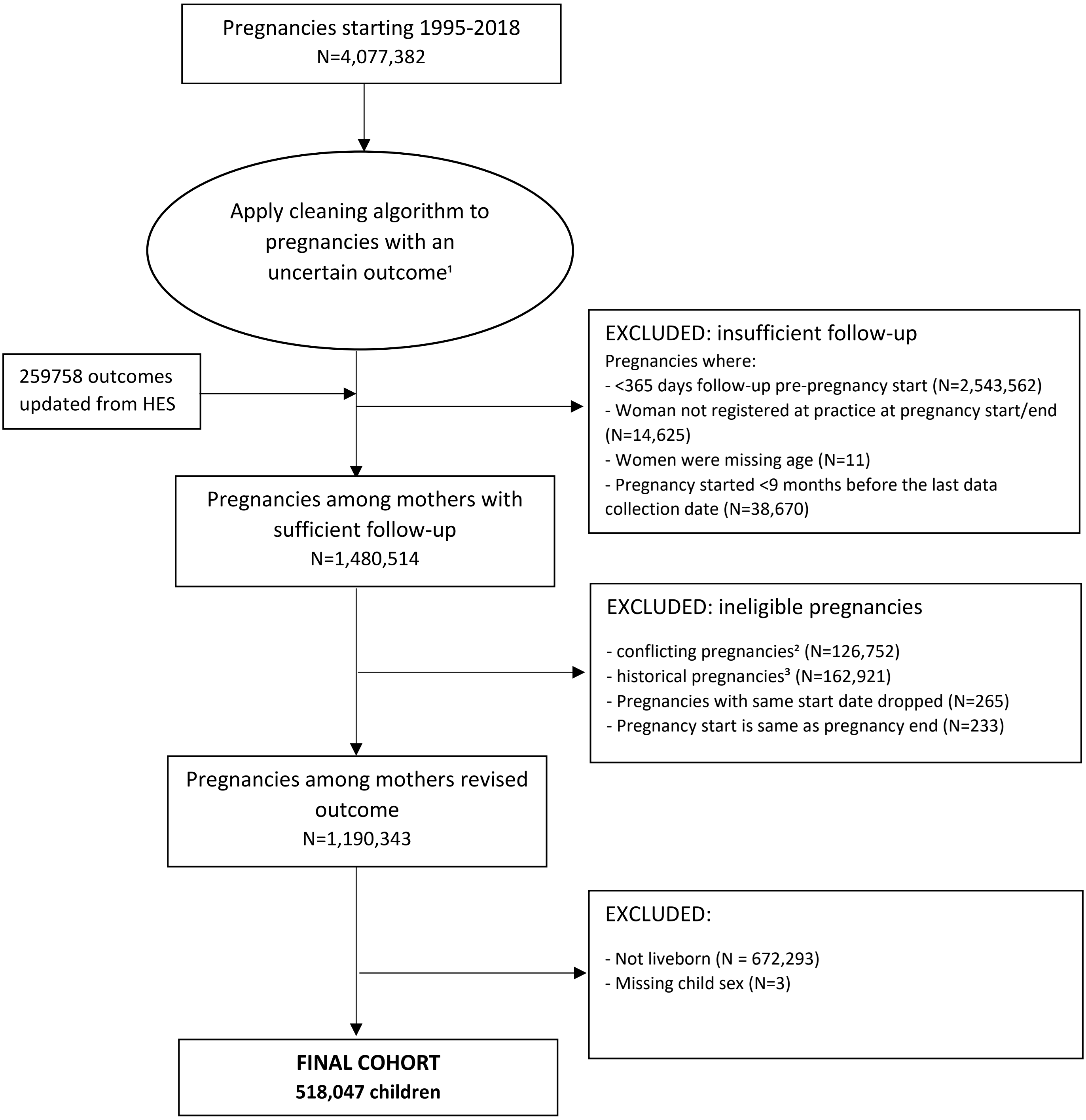

[1] See supplementary methods for more information on how we dealt with Uncertain pregnancies in the CPRD Pregnancy Register
[2]Conflicting pregnancies refer to pregnancies where dates overlap.
[3]Historical pregnancies refer to past pregnancies recorded at a later date

Figure S2 – Flowchart of cohort derivation for DOHaD (Sweden)

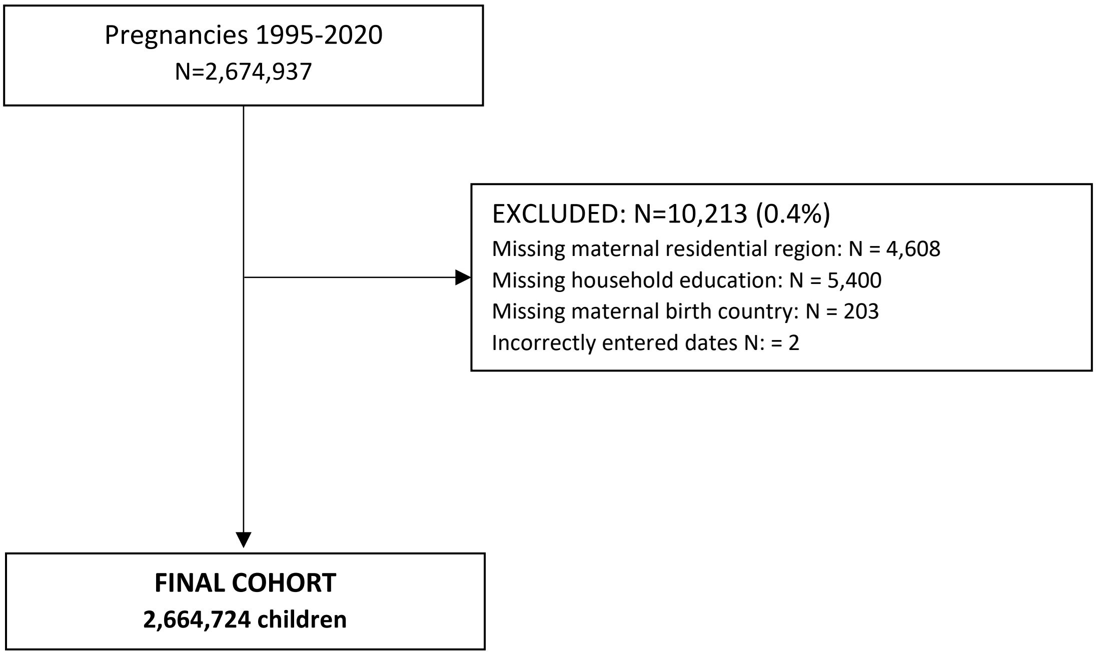

Figure S3 – Primary Analysis: Pooled risk difference results relative to no ASM at age 12

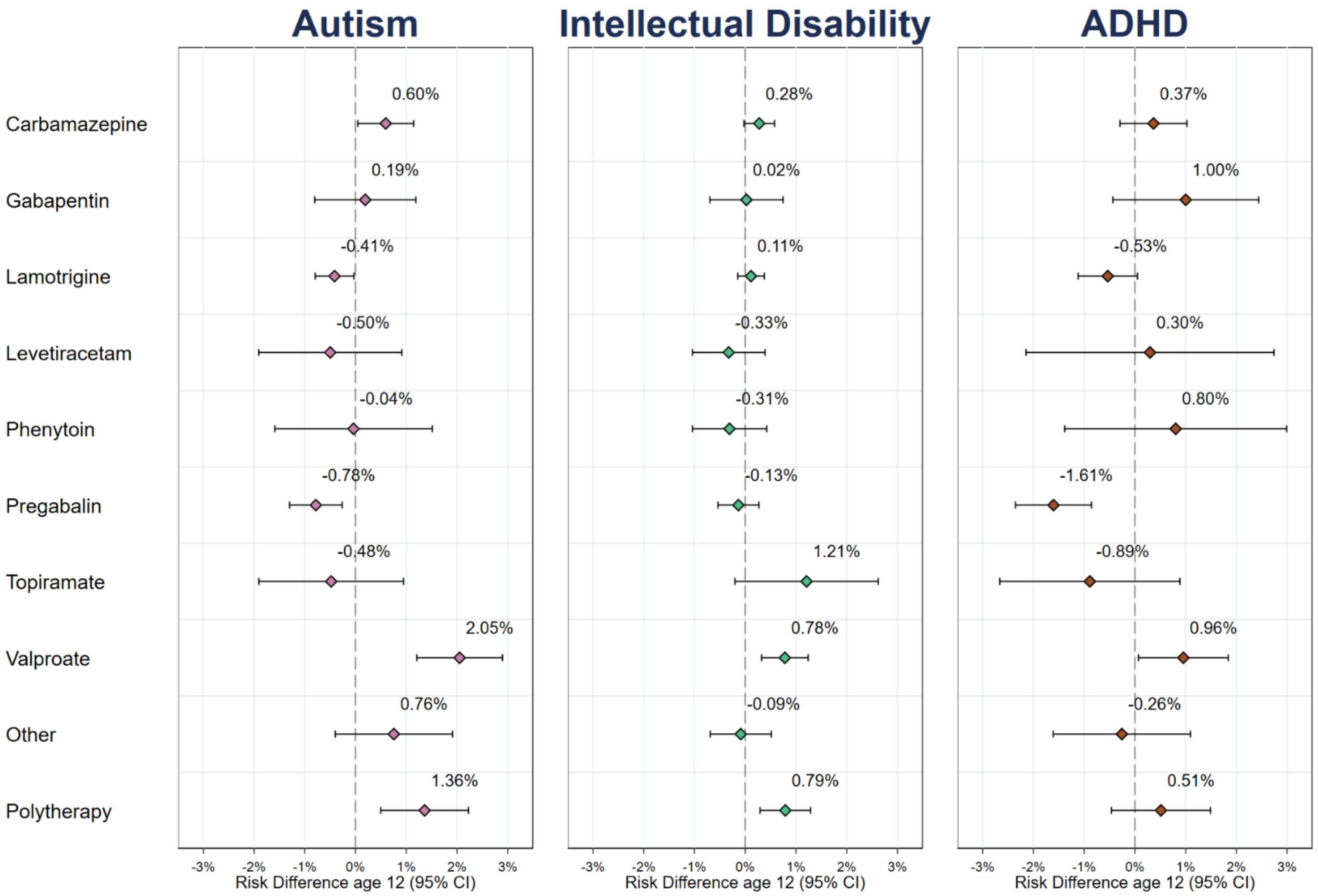

Legend: Data are presented as pooled absolute adjusted risk difference +/- 95% confidence limits, estimated using fixed-effects meta-analysis on the log-risk scale. All estimates are adjusted for maternal age, region, diagnosis of neurodevelopmental conditions before pregnancy, evidence of hazardous drinking and illicit drug use during pregnancy, gravidity, health care utilization, seizure events, use of antipsychotics and antidepressants in the year prior to pregnancy, vomiting or antiemetic prescriptions during pregnancy, and socioeconomic position. Sample size for figure: Carbamazepine = 3030, Gabapentin = 1428, Lamotrigine = 5974, Levetiracetam = 806, Phenytoin = 240, Pregabalin = 1715, Topiramate = 418, Valproate = 1601, Other ASM = 543, Polytherapy = 1740. Source data are provided as a Source Data file. Abbreviations: ADHD = Attention deficit hyperactivity disorder, ASM = Antiseizure medication.

Figure S4 – Primary Analysis: Country specific risk difference results for autism relative to no ASM

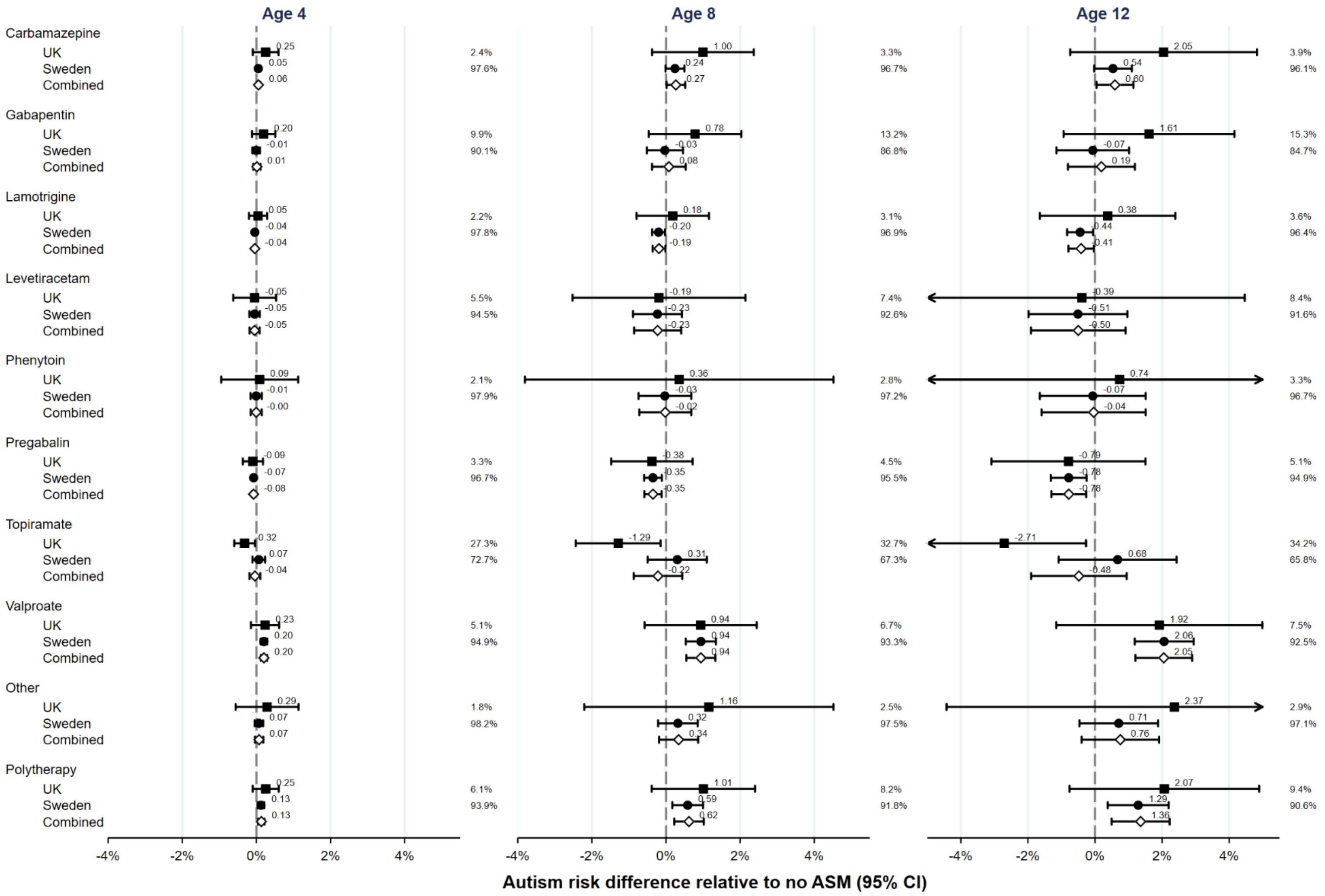

Legend: Data are presented as absolute adjusted risk difference +/- 95% confidence limits for autism, combined using fixed-effects meta-analysis on the log-risk scale. All estimates are adjusted for maternal age, region, diagnosis of neurodevelopmental conditions before pregnancy, evidence of hazardous drinking and illicit drug use during pregnancy, gravidity, health care utilization, seizure events, use of antipsychotics and antidepressants in the year prior to pregnancy, vomiting or antiemetic prescriptions during pregnancy, and socioeconomic position. Combined sample size: Carbamazepine = 3030, Gabapentin = 1428, Lamotrigine = 5974, Levetiracetam = 806, Phenytoin = 240, Pregabalin = 1715, Topiramate = 418, Valproate = 1601, Other ASM = 543, Polytherapy = 1740. Source data are provided as a Source Data file. Abbreviations: ASM = Antiseizure medication.

Figure S5 – Primary Analysis: Country specific risk difference results for intellectual disability relative to no ASM

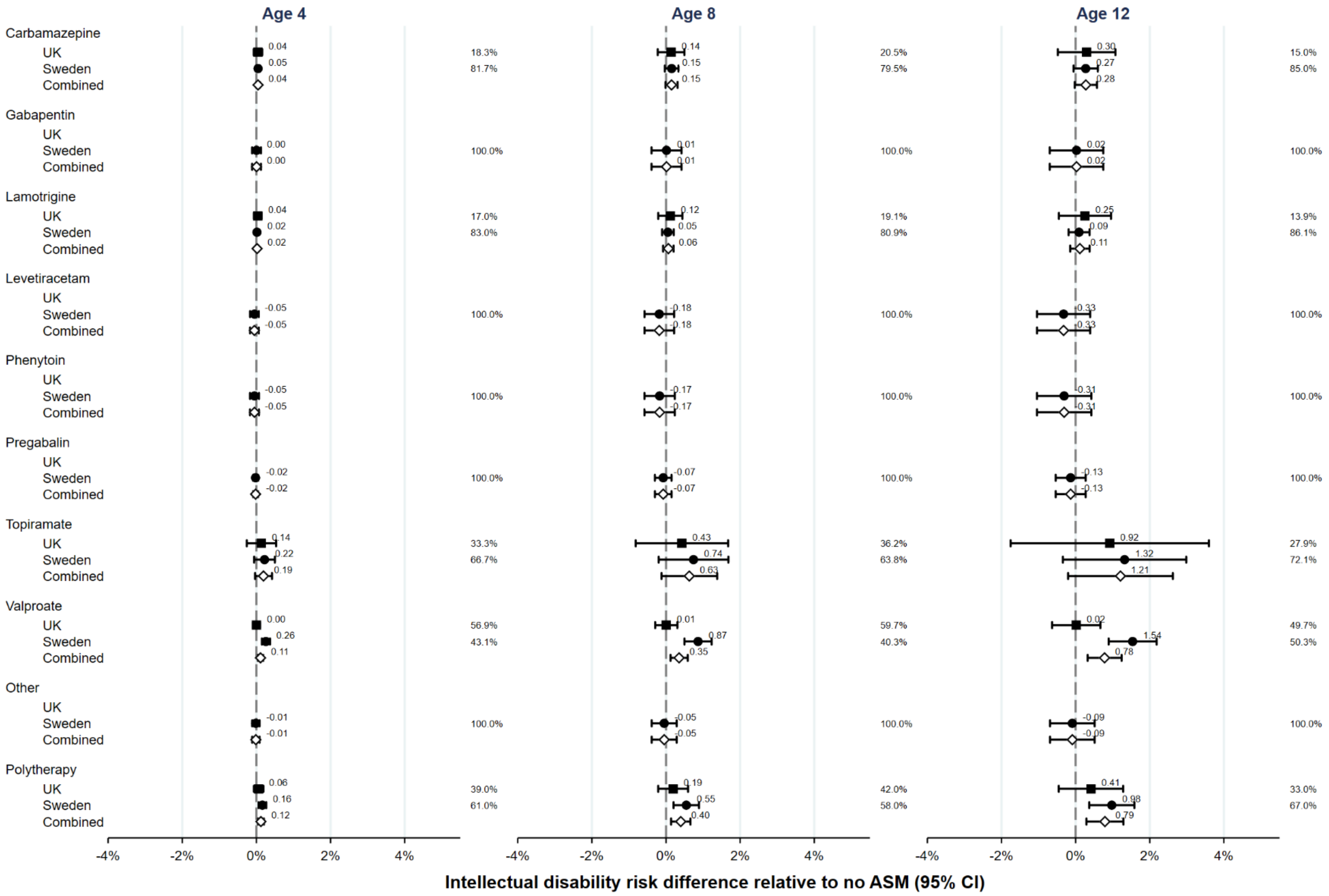

Legend: Data are presented as absolute adjusted risk difference +/- 95% confidence limits for intellectual disability, combined using fixed-effects meta-analysis on the log-risk scale. All estimates are adjusted for maternal age, region, diagnosis of neurodevelopmental conditions before pregnancy, evidence of hazardous drinking and illicit drug use during pregnancy, gravidity, health care utilization, seizure events, use of antipsychotics and antidepressants in the year prior to pregnancy, vomiting or antiemetic prescriptions during pregnancy, and socioeconomic position. Combined sample size: Carbamazepine = 3030, Gabapentin = 1428, Lamotrigine = 5974, Levetiracetam = 806, Phenytoin = 240, Pregabalin = 1715, Topiramate = 418, Valproate = 1601, Other ASM = 543, Polytherapy = 1740. Source data are provided as a Source Data file. Abbreviations: ASM = Antiseizure medication.

Figure S6 – Primary Analysis: Country specific risk difference results for ADHD relative to no ASM

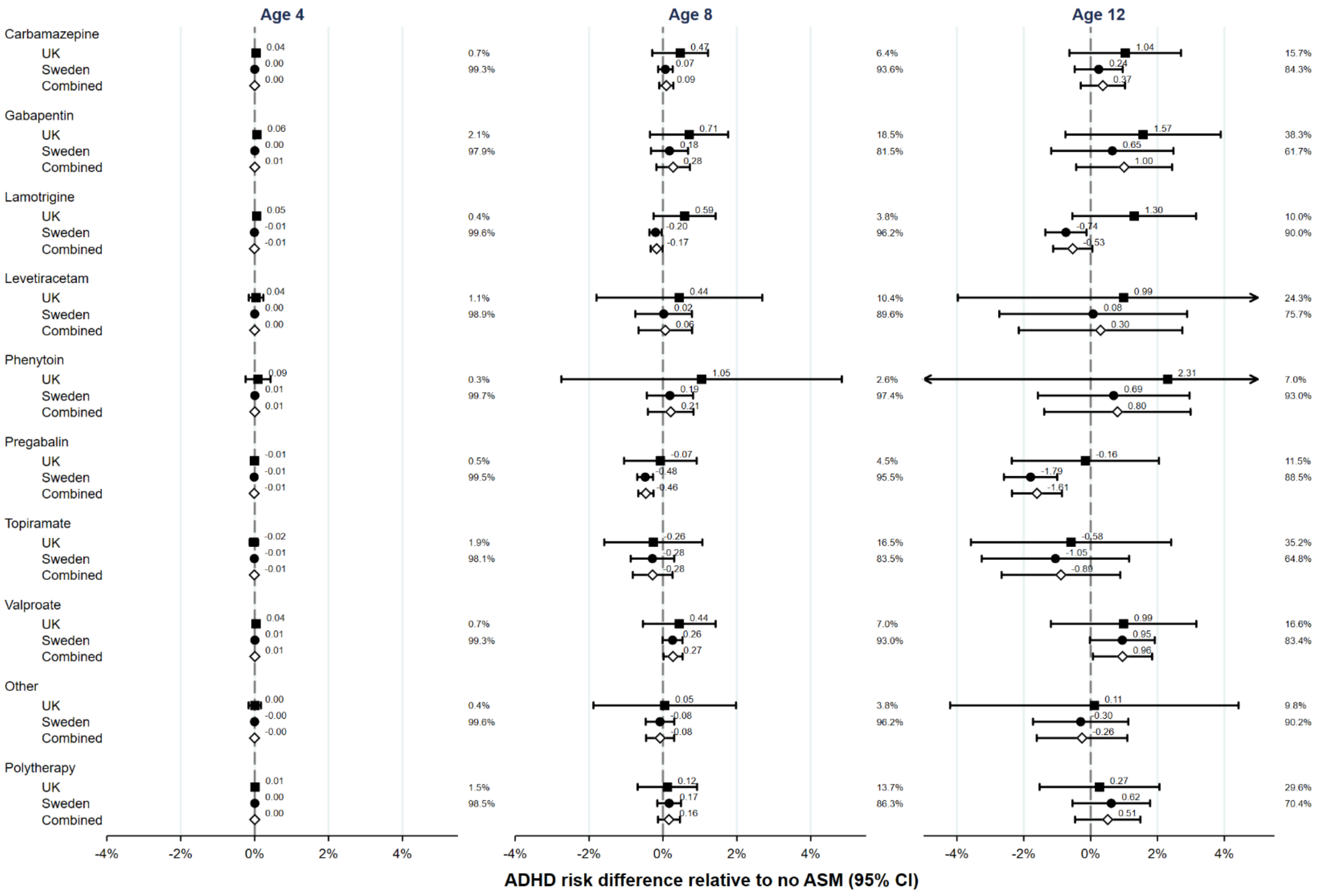

Legend: Data are presented as absolute adjusted risk difference +/- 95% confidence limits for ADHD, combined using fixed-effects meta-analysis on the log-risk scale. All estimates are adjusted for maternal age, region, diagnosis of neurodevelopmental conditions before pregnancy, evidence of hazardous drinking and illicit drug use during pregnancy, gravidity, health care utilization, seizure events, use of antipsychotics and antidepressants in the year prior to pregnancy, vomiting or antiemetic prescriptions during pregnancy, and socioeconomic position. Combined sample size: Carbamazepine = 3030, Gabapentin = 1428, Lamotrigine = 5974, Levetiracetam = 806, Phenytoin = 240, Pregabalin = 1715, Topiramate = 418, Valproate = 1601, Other ASM = 543, Polytherapy = 1740. Source data are provided as a Source Data file. Abbreviations: ADHD = Attention deficit hyperactivity disorder, ASM = Antiseizure medication.

Figure S7 – Primary Analysis: Country specific hazard ratio results

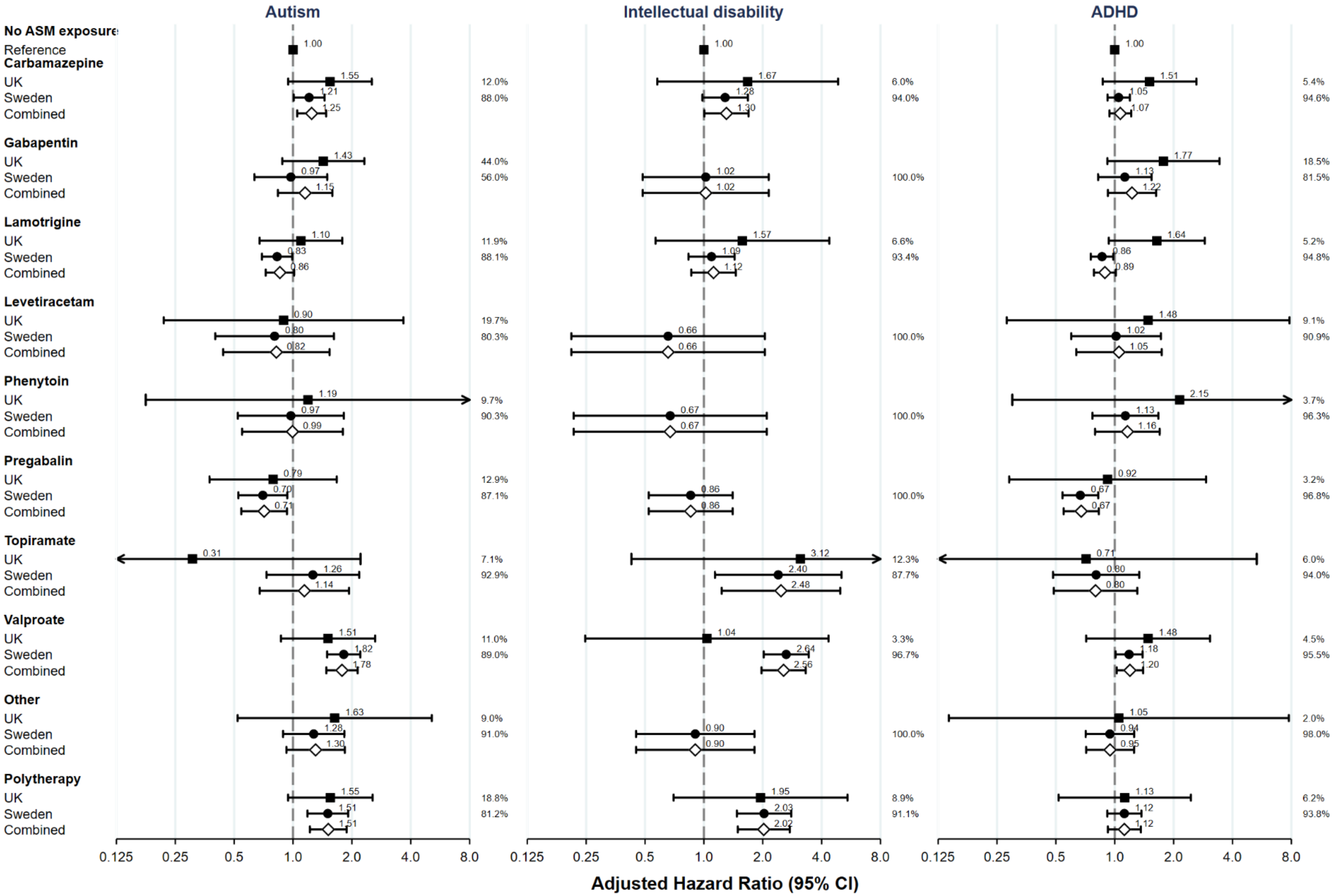

Legend: Data are presented as hazard ratios +/- 95% confidence limits for autism, intellectual disability, and ADHD, combined using fixed-effects meta-analysis on the log-hazard scale. All estimates are adjusted for maternal age, region, diagnosis of neurodevelopmental conditions before pregnancy, evidence of hazardous drinking and illicit drug use during pregnancy, gravidity, health care utilization, seizure events, use of antipsychotics and antidepressants in the year prior to pregnancy, vomiting or antiemetic prescriptions during pregnancy, and socioeconomic position. Combined sample size: Carbamazepine = 3030, Gabapentin = 1428, Lamotrigine = 5974, Levetiracetam = 806, Phenytoin = 240, Pregabalin = 1715, Topiramate = 418, Valproate = 1601, Other ASM = 543, Polytherapy = 1740. Source data are provided as a Source Data file.
Abbreviations: ADHD = Attention deficit hyperactivity disorder, ASM = Antiseizure medication.

Figure S8 – Discordant Sibling Analysis: Country specific within-family hazard ratio results

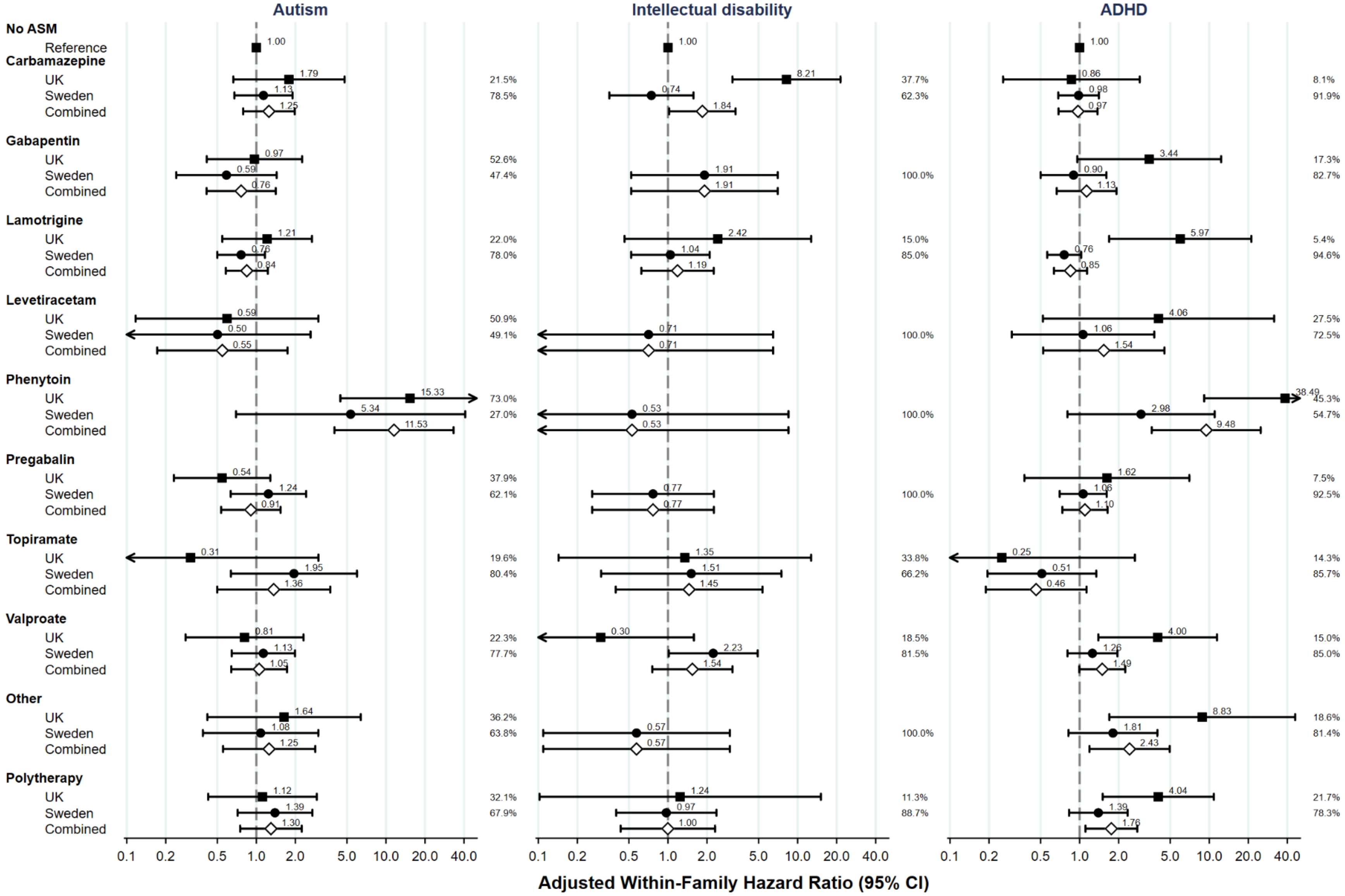

Legend: Data are presented as within-family hazard ratios +/- 95% confidence limits for autism, intellectual disability, and ADHD, combined using fixed-effects meta-analysis on the log-hazard scale. All estimates are adjusted for the same covariates as the primary analysis to the extent that they vary across siblings. Source data are provided as a Source Data file. Abbreviations: ADHD = Attention deficit hyperactivity disorder, ASM = Antiseizure medication.

Figure S9 – Active Comparator Analysis: Country specific hazard ratio results

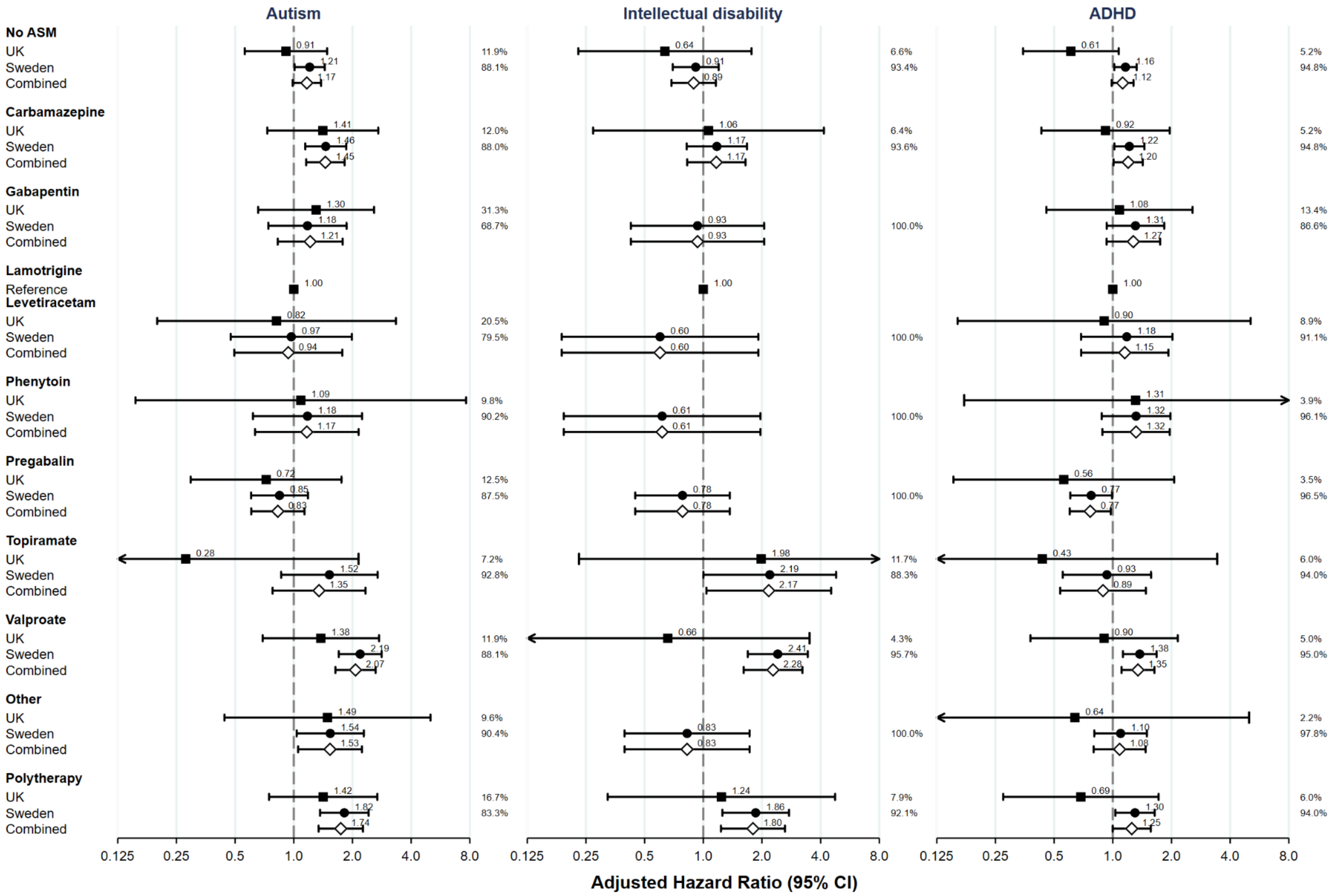

Legend: Data are presented as hazard ratios +/- 95% confidence limits for autism, intellectual disability, and ADHD, combined using fixed-effects meta-analysis on the log-hazard scale, using lamotrigine as the referent. All estimates are adjusted for the same covariates as the primary analysis. Source data are provided as a Source Data file. Abbreviations: ADHD = Attention deficit hyperactivity disorder, ASM = Antiseizure medication.

Figure S10 – Indication Stratified Analysis: Pooled adjusted marginal risk at age 12 results

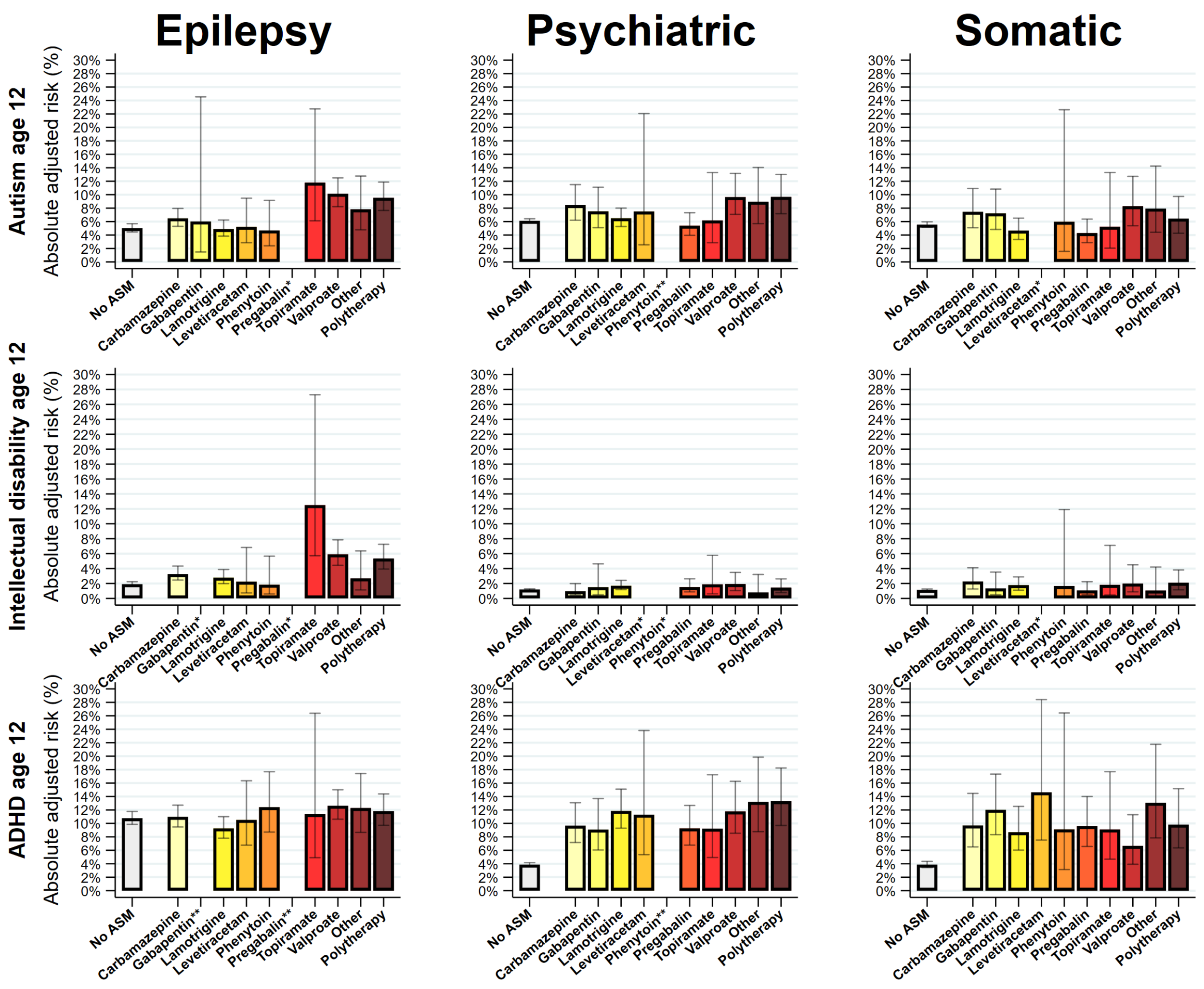

Legend: Data are presented as pooled absolute adjusted risk by age 12, +/- 95% confidence limits, for autism, intellectual disability, and ADHD, combined using fixed-effects meta-analysis on the log-hazard scale, among those with a psychiatric indication. All estimates are adjusted for the same covariates as the primary analysis. * Unable to estimate due to 0 exposed case counts across both cohorts (See Table S10). ** Estimate not presented as it falls outside the plot region; tote that this is due to a small number of exposed cases (See Table S11 and S12). Note that pooled estimates of the risk of ADHD among those not exposed to an ASM are heavily weighted towards the UK (CPRD) for psychiatric and somatic indications, where a lower risk was estimated than in Sweden (DOHaD), due to the calculation of lower standard errors for estimated risks closer to 0.. Source data are provided as a Source Data file. Abbreviations: ADHD = Attention deficit hyperactivity disorder, ASM = Antiseizure medication.

Figure S11 – Indication Stratified Analysis: Pooled hazard ratio results

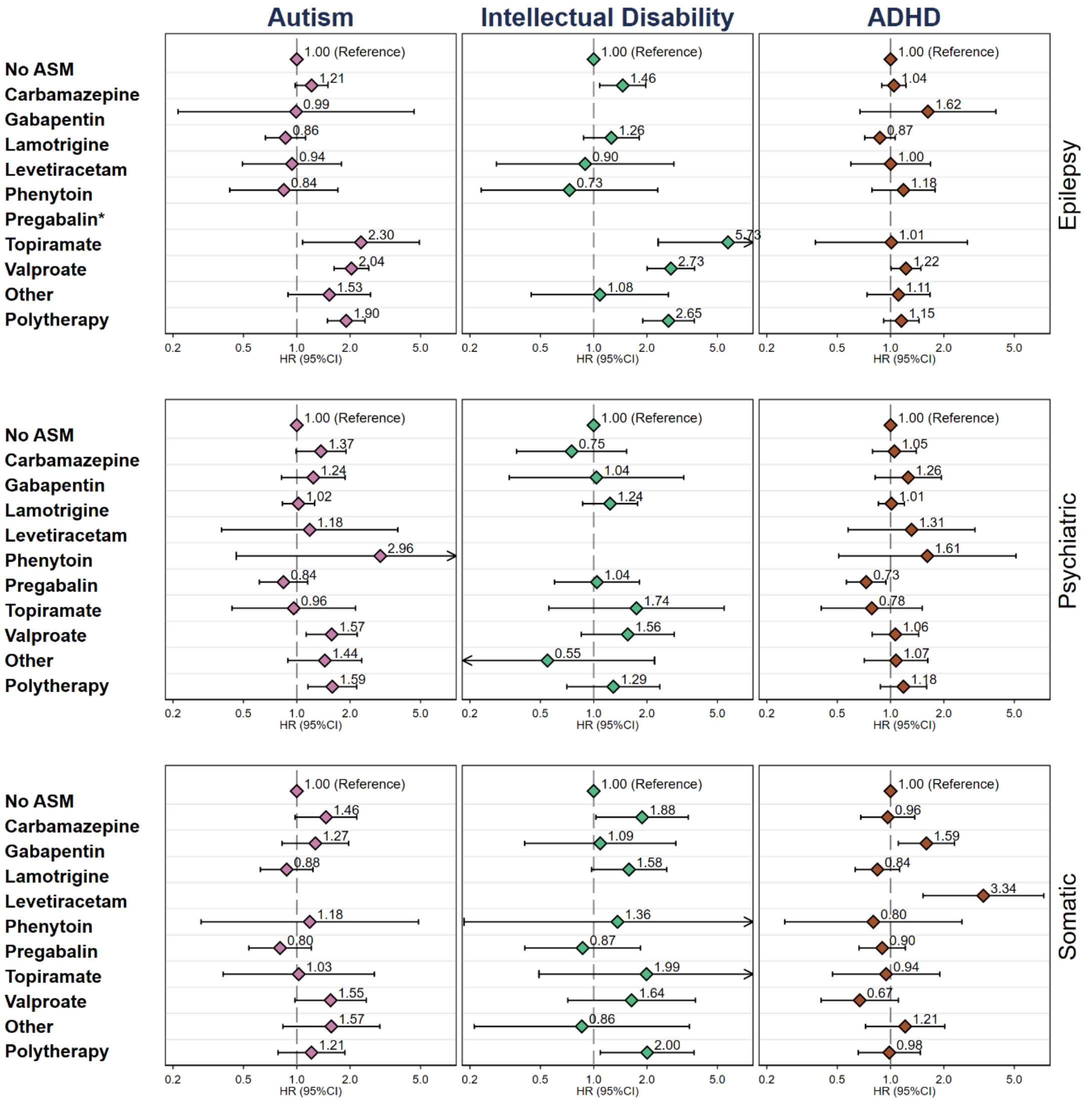

Legend: Data are presented as hazard ratios +/- 95% confidence limits for autism, intellectual disability, and ADHD, combined using fixed-effects meta-analysis on the log-hazard scale, among those with a psychiatric indication. All estimates are adjusted for the same covariates as the primary analysis. *Pregabalin has been removed from the plot for epilepsy and ADHD as the estimates fall outside the plot region, note that this is due to a small number of exposed cases contributed by CPRD only (see Table 2 & S10). Source data are provided as a Source Data file. Abbreviations: ADHD = Attention deficit hyperactivity disorder, ASM = Antiseizure medication.

Figure S12 – Indication Stratified Analysis: Country specific hazard ratio results for epilepsy

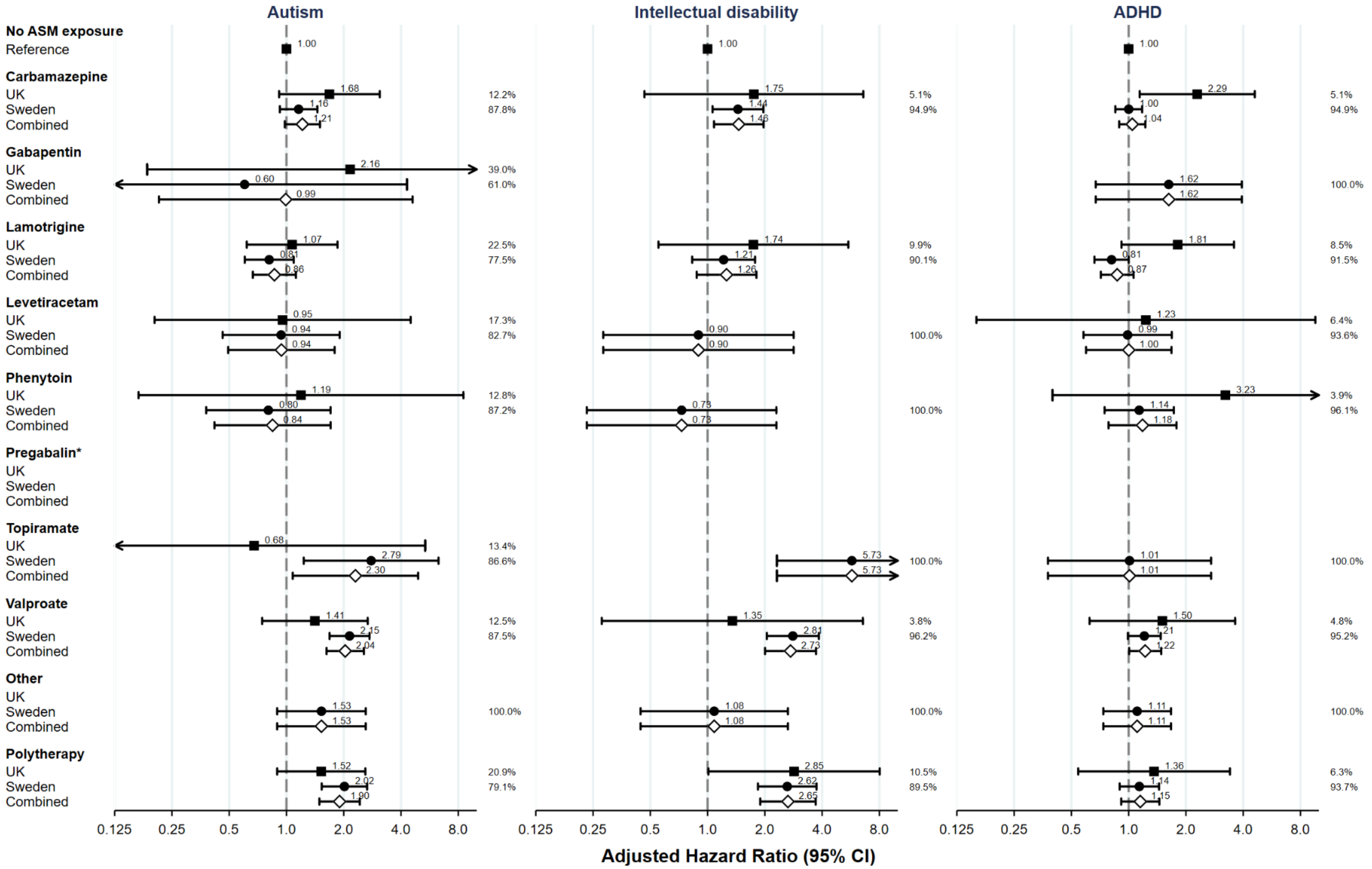

Legend: Data are presented as hazard ratios +/- 95% confidence limits for autism, intellectual disability, and ADHD, combined using fixed-effects meta-analysis on the log-hazard scale, among those with a psychiatric indication. All estimates are adjusted for the same covariates as the primary analysis. *Pregabalin has been removed from the plot for ADHD as the estimates fall outside the plot region, note that this is due to a small number of exposed cases contributed by CPRD only (see Table 2 & S10). Source data are provided as a Source Data file. Abbreviations: ADHD = Attention deficit hyperactivity disorder, ASM = Antiseizure medication.

Figure S13 – Indication Stratified Analysis: Country specific hazard ratio results for psychiatric indications

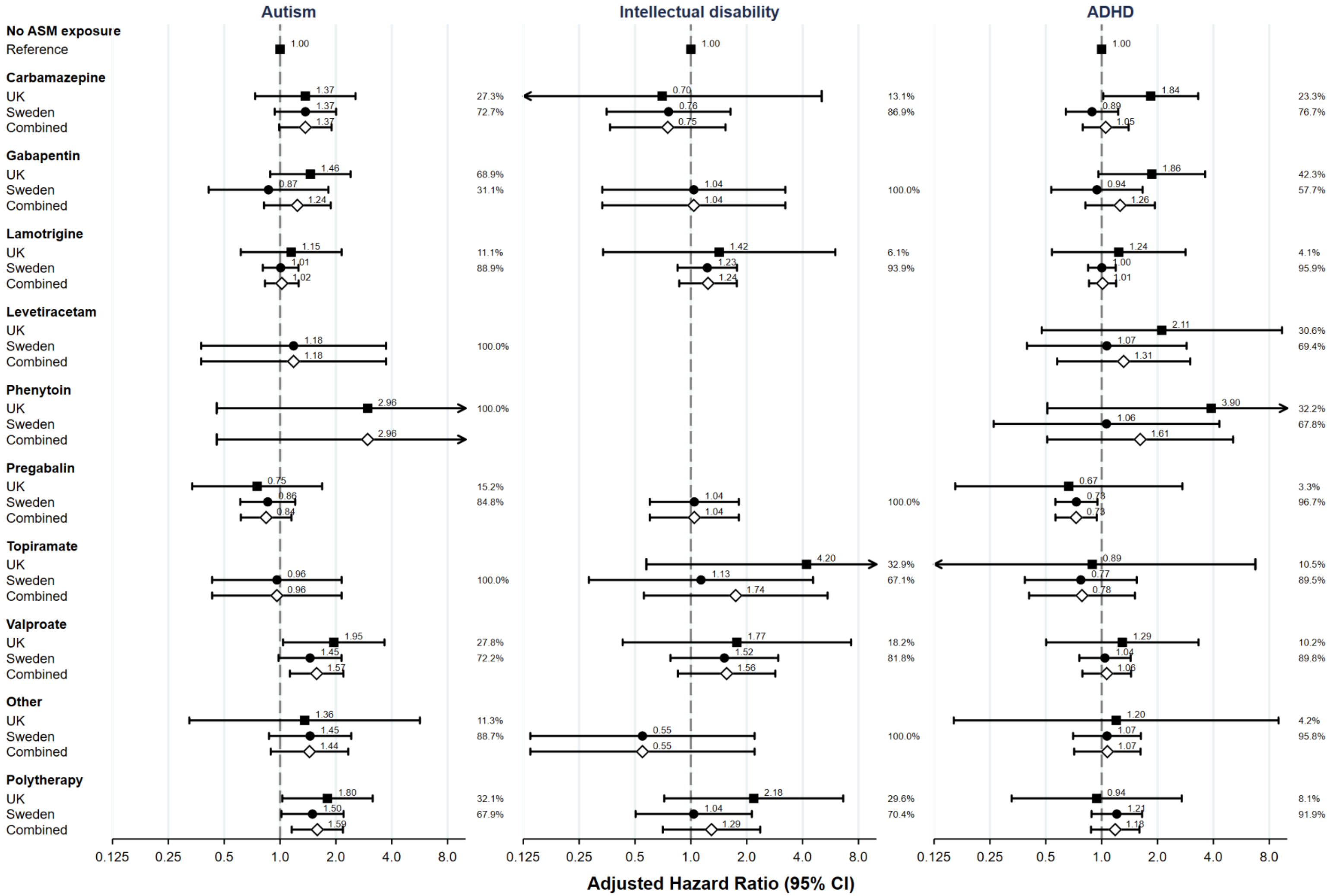

Legend: Data are presented as hazard ratios +/- 95% confidence limits for autism, intellectual disability, and ADHD, combined using fixed-effects meta-analysis on the log-hazard scale, among those with a psychiatric indication. All estimates are adjusted for the same covariates as the primary analysis. Source data are provided as a Source Data file. Abbreviations: ADHD = Attention deficit hyperactivity disorder, ASM = Antiseizure medication.

Figure S14 – Indication Stratified Analysis: Country specific hazard ratio results for somatic indications

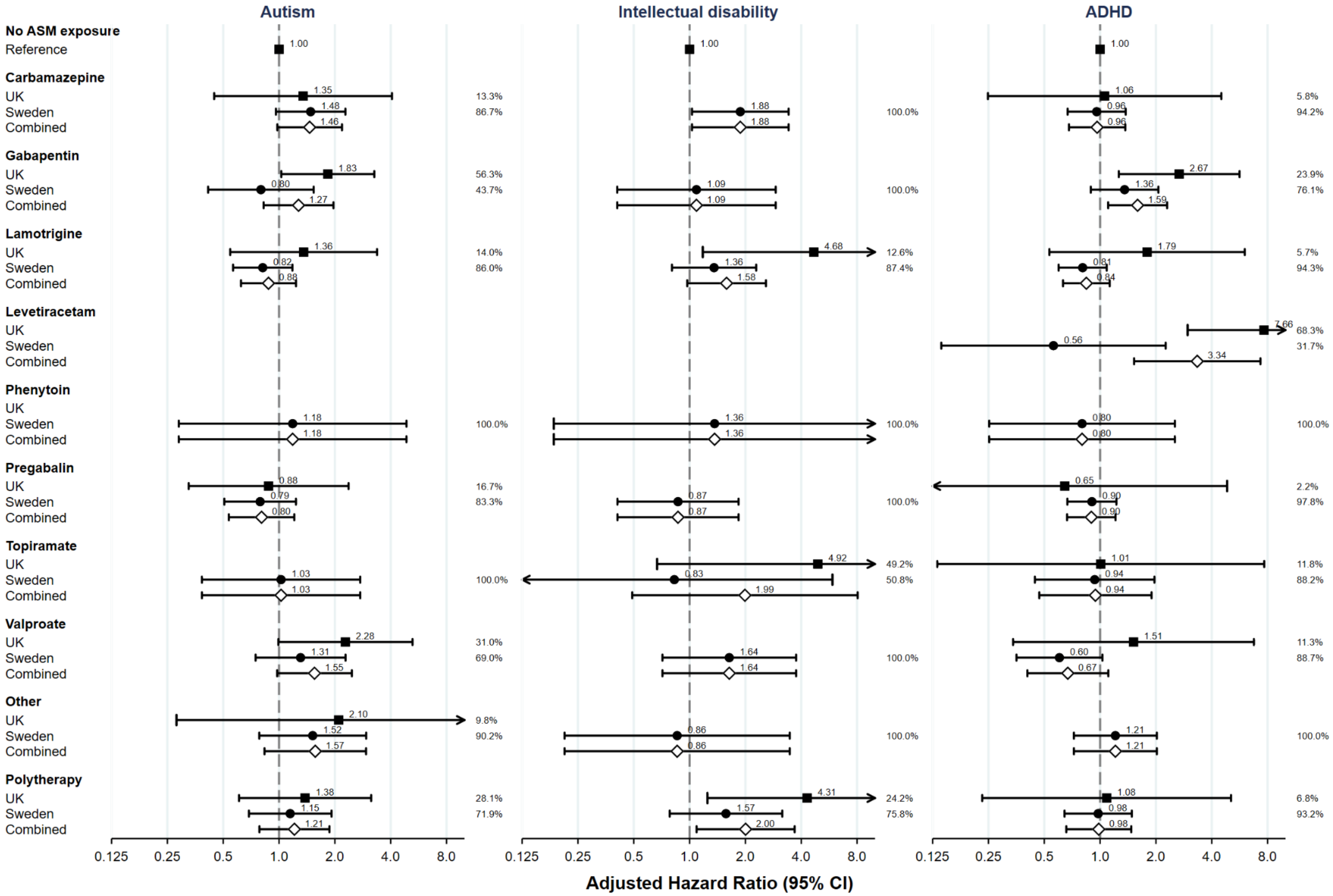

Legend: Data are presented as hazard ratios +/- 95% confidence limits for autism, intellectual disability, and ADHD, combined using fixed-effects meta-analysis on the log-hazard scale, among those with a somatic indication. All estimates are adjusted for the same covariates as the primary analysis. Source data are provided as a Source Data file. Abbreviations: ADHD = Attention deficit hyperactivity disorder, ASM = Antiseizure medication.

References

1. Daugaard CA, Pedersen L, Sun Y, Dreier JW, Christensen J. Association of Prenatal Exposure to Valproate and Other Antiepileptic Drugs With Intellectual Disability and Delayed Childhood Milestones. *JAMA Netw Open*. Nov 2 2020;3(11):e2025570. doi:10.1001/jamanetworkopen.2020.25570

2. Bjørk M-H, Zoega H, Leinonen MK, et al. Association of Prenatal Exposure to Antiseizure Medication With Risk of Autism and Intellectual Disability. *JAMA Neurology*. 2022;79(7):672-681. doi:10.1001/jamaneurol.2022.1269 %J JAMA Neurology

3. Dreier JW, Bjork MH, Alvestad S, et al. Prenatal Exposure to Antiseizure Medication and Incidence of Childhood- and Adolescence-Onset Psychiatric Disorders. *JAMA Neurol*. Apr 17 2023;doi:10.1001/jamaneurol.2023.0674

4. Ren T, Lee PMY, Li F, Li J. Prenatal Carbamazepine Exposure and Academic Performance in Adolescents: A Population-Based Cohort Study. *Neurology*. Feb 14 2023;100(7):e728-e738. doi:10.1212/WNL.0000000000201529

5. Herrett E, Gallagher AM, Bhaskaran K, et al. Data Resource Profile: Clinical Practice Research Datalink (CPRD). *Int J Epidemiol*. Jun 2015;44(3):827-36. doi:10.1093/ije/dyv098

6. Chisholm J. The Read clinical classification. *BMJ*. Apr 28 1990;300(6732):1092. doi:10.1136/bmj.300.6732.1092

7. Clinical Practice Research Datalink. Data from: CPRD GOLD August 2021. 2021;2021.08.001. doi:https://doi.org/10.48329/dfcr-ap05

8. Minassian C, Williams R, Meeraus WH, Smeeth L, Campbell OMR, Thomas SL. Methods to generate and validate a Pregnancy Register in the UK Clinical Practice Research Datalink primary care database. *Pharmacoepidemiol Drug Saf*. Jul 2019;28(7):923-933. doi:10.1002/pds.4811

9. CPRD Mother Baby Link Documentation (Version 1.2) (2017).

10. Clinical Practice Research Datalink. Data from: CPRD GOLD HES APC March 2021. 2021;2021.03.001. doi:https://doi.org/10.48329/aven-n539

11. Clinical Practice Research Datalink. Data from: CPRD GOLD HES OP August 2021. 2021;2021.08.001. doi:https://doi.org/10.48329/cp5e-7790

12. Clinical Practice Research Datalink. Data from: CPRD GOLD HES A&E August 2021. 2021;2021.08.001. doi:https://doi.org/10.48329/XTWG-GP32

13. Clinical Practice Research Datalink. Data from: CPRD GOLD Small Area data (patient) March 2021. 2021;2021.03.001. doi:https://doi.org/10.48329/1qnt-r031

14. Clinical Practice Research Datalink. Data from: CPRD GOLD Small Area data (practice) March 2021. 2021;2021.03.001. doi:https://doi.org/10.48329/g915-nk16

15. Clinical Practice Research Datalink. Data from: CPRD ONS deaths March 2021. 2021;2021.03.001. doi:https://doi.org/10.48329/4c3r-av86

16. Ahlqvist VH, Sjöqvist H, Dalman C, et al. Acetaminophen Use During Pregnancy and Children's Risk of Autism, ADHD, and Intellectual Disability. *JAMA*. 2024;331(14):1205-1214. doi:10.1001/jama.2024.3172 %J JAMA

17. Ludvigsson JF, Otterblad-Olausson P, Pettersson BU, Ekbom A. The Swedish personal identity number: possibilities and pitfalls in healthcare and medical research. *Eur J Epidemiol*. 2009;24(11):659-67. doi:10.1007/s10654-009-9350-y

18. Cnattingius S, Kallen K, Sandstrom A, et al. The Swedish medical birth register during five decades: documentation of the content and quality of the register. *Eur J Epidemiol*. Jan 2023;38(1):109-120. doi:10.1007/s10654-022-00947-5

19. Wettermark B, Hammar N, Fored CM, et al. The new Swedish Prescribed Drug Register--opportunities for pharmacoepidemiological research and experience from the first six months. *Pharmacoepidemiol Drug Saf*. Jul 2007;16(7):726-35. doi:10.1002/pds.1294

20. Ludvigsson JF, Andersson E, Ekbom A, et al. External review and validation of the Swedish national inpatient register. *BMC public health*. Jun 9 2011;11:450. doi:10.1186/1471-2458-11-450

21. Statistics Sweden. Longitudinal integration database for health insurance and labour market studies (LISA by Swedish acronym). English; 2012.
22. Campbell J, Bhaskaran K, Thomas S, Williams R, McDonald HI, Minassian C. Investigating the optimal handling of uncertain pregnancy episodes in the CPRD GOLD Pregnancy Register: a methodological study using UK primary care data. *BMJ Open*. Feb 22 2022;12(2):e055773. doi:10.1136/bmjopen-2021-055773
