# Supplementary material for: Antiseizure medication use during pregnancy and children’s neurodevelopmental outcomes
Source: Nat Commun. 2024 Nov 15;15:9640. doi: 10.1038/s41467-024-53813-1 (PMC11568279; doi:10.1038/s41467-024-53813-1)
Supplement: Supplementary file 2 — Reporting Summary [file 41467_2024_53813_MOESM2_ESM.pdf]

Corresponding author(s): Viktor H. Ahlqvist

Last updated by author(s): Oct 15, 2024

Reporting Summary

Nature Portfolio wishes to improve the reproducibility of the work that we publish. This form provides structure for consistency and transparency in reporting. For further information on Nature Portfolio policies, see our Editorial Policies and the Editorial Policy Checklist.

Statistics

For all statistical analyses, confirm that the following items are present in the figure legend, table legend, main text, or Methods section.

n/a	Confirmed	
☐	☒	The exact sample size (*n*) for each experimental group/condition, given as a discrete number and unit of measurement
☐	☒	A statement on whether measurements were taken from distinct samples or whether the same sample was measured repeatedly
☐	☒	The statistical test(s) used AND whether they are one- or two-sided<br>*Only common tests should be described solely by name; describe more complex techniques in the Methods section.*
☐	☒	A description of all covariates tested
☐	☒	A description of any assumptions or corrections, such as tests of normality and adjustment for multiple comparisons
☐	☒	A full description of the statistical parameters including central tendency (e.g. means) or other basic estimates (e.g. regression coefficient) AND variation (e.g. standard deviation) or associated estimates of uncertainty (e.g. confidence intervals)
☐	☒	For null hypothesis testing, the test statistic (e.g. *F*, *t*, *r*) with confidence intervals, effect sizes, degrees of freedom and *P* value noted<br>*Give P values as exact values whenever suitable.*
☒	☐	For Bayesian analysis, information on the choice of priors and Markov chain Monte Carlo settings
☒	☐	For hierarchical and complex designs, identification of the appropriate level for tests and full reporting of outcomes
☒	☐	Estimates of effect sizes (e.g. Cohen's *d*, Pearson's *r*), indicating how they were calculated

Our web collection on statistics for biologists contains articles on many of the points above.

Software and code

Policy information about availability of computer code

Data collection	Data preparation and derivation code can be made available under relevant approvals.
Data analysis	Analysis scripts and code lists can be found at https://github.com/pmadleydowd/PREPArE-ASM-Neurodevelopment.

For manuscripts utilizing custom algorithms or software that are central to the research but not yet described in published literature, software must be made available to editors and reviewers. We strongly encourage code deposition in a community repository (e.g. GitHub). See the Nature Portfolio guidelines for submitting code & software for further information.

Data

Policy information about availability of data

All manuscripts must include a data availability statement. This statement should provide the following information, where applicable:

- Accession codes, unique identifiers, or web links for publicly available datasets
- A description of any restrictions on data availability
- For clinical datasets or third party data, please ensure that the statement adheres to our policy

Swedish privacy law prohibits us from making registry data publicly available. The data supporting our findings were used under license and ethical approval for the current study. Readers interested in obtaining microdata or replicating our study may seek similar approvals and inquire through Statistics Sweden. For further advice see: https://www.scb.se/en/services/guidance-for-researchers-and-universities/, or contact Statistics Sweden at: mikrodata@scb.se.

Access to CPRD data can be obtained by following guidance at https://cprd.com/how-access-cprd-data.

Research involving human participants, their data, or biological material

Policy information about studies with human participants or human data. See also policy information about sex, gender (identity/presentation), and sexual orientation and race, ethnicity and racism.

Reporting on sex and gender	Gender data has not been collected. All language is with reference to sex and/or individuals of childbearing potential.
Reporting on race, ethnicity, or other socially relevant groupings	In Sweden, we controlled for country of birth, since there are well-described inequalities in access to pregnancy care in the country
Population characteristics	All details are available in Table 1 and the supplementary material.
Recruitment	All individuals available in the relevant electronic health data sources were included.
Ethics oversight	The Swedish Ethical Review Authority (DNR 2020-05516) gave ethical approval for the Swedish analysis, and CPRD approved the UK analysis (eRAP application: 20_000228).

Note that full information on the approval of the study protocol must also be provided in the manuscript.

Field-specific reporting

Please select the one below that is the best fit for your research. If you are not sure, read the appropriate sections before making your selection.

☒ Life sciences ☐ Behavioural & social sciences ☐ Ecological, evolutionary & environmental sciences

For a reference copy of the document with all sections, see nature.com/documents/nr-reporting-summary-flat.pdf

Life sciences study design

All studies must disclose on these points even when the disclosure is negative.

Sample size	No sample-size calculations were performed, as all eligible individuals in the respective data were included
Data exclusions	We excluded 3 children who had missing sex information. Details on how missing prescription information were dealt with are provided in the section titled "Prescription data cleaning procedure – CPRD (UK)". Following the data cleaning procedure for prescriptions, the exposure, outcome and all covariates selected for adjustment in models were complete.<br><br>Sweden: Pregnancies where information was missing on maternal residential region (N=4,608), household education (N=5,400), or maternal country of birth (N=203), and 2 pregnancies with infeasible dates were excluded from the cohort. In total 10,213 individuals (0.4% of the cohort) were excluded for missing data in any of these covariates.
Replication	No external attempts to replicate the findings were performed.
Randomization	No randomization was performed due to the observational nature of the study. Imbalances in covariates were accounted for through regression modeling.
Blinding	No blinding was conducted as the study relied on available data.

Reporting for specific materials, systems and methods

We require information from authors about some types of materials, experimental systems and methods used in many studies. Here, indicate whether each material, system or method listed is relevant to your study. If you are not sure if a list item applies to your research, read the appropriate section before selecting a response.

Materials & experimental systems

n/a	Involved in the study
☒	☐ Antibodies
☒	☐ Eukaryotic cell lines
☒	☐ Palaeontology and archaeology
☒	☐ Animals and other organisms
☒	☐ Clinical data
☒	☐ Dual use research of concern
☒	☐ Plants

Methods

n/a	Involved in the study
☒	☐ ChIP-seq
☒	☐ Flow cytometry
☒	☐ MRI-based neuroimaging

Plants

Seed stocks

Report on the source of all seed stocks or other plant material used. If applicable, state the seed stock centre and catalogue number. If plant specimens were collected from the field, describe the collection location, date and sampling procedures.

Novel plant genotypes

Describe the methods by which all novel plant genotypes were produced. This includes those generated by transgenic approaches, gene editing, chemical/radiation-based mutagenesis and hybridization. For transgenic lines, describe the transformation method, the number of independent lines analyzed and the generation upon which experiments were performed. For gene-edited lines, describe the editor used, the endogenous sequence targeted for editing, the targeting guide RNA sequence (if applicable) and how the editor was applied.

Authentication

Describe any authentication procedures for each seed stock used or novel genotype generated. Describe any experiments used to assess the effect of a mutation and, where applicable, how potential secondary effects (e.g. second site T-DNA insertions, mosiacism, off-target gene editing) were examined.
